# Supplementary material for: Evolutionary dynamics and geographic dispersal of beta coronaviruses in African bats
Source: PeerJ. 2020 Nov 26;8:e10434. doi: 10.7717/peerj.10434 (PMC7700737; doi:10.7717/peerj.10434)
Supplement: Supplemental Information 1 — Supplementary Figure 1. Root to tip regression analysis plots of the genetic distances against the year of isolation for the Bt-CoV sequences analyzed in this study. Figure 2a represents Afr-Bt-CoV, while 2b represents sequences of African, Asian and European origins. [file peerj-08-10434-s001.pdf]

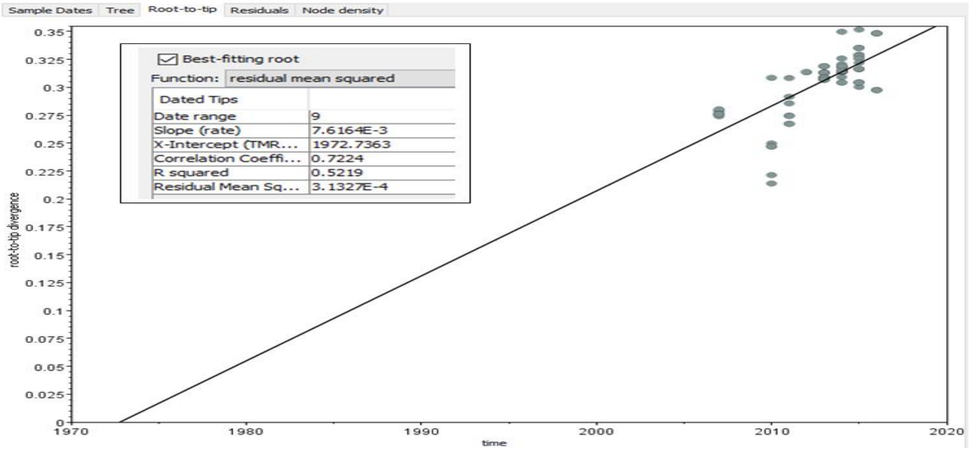

A

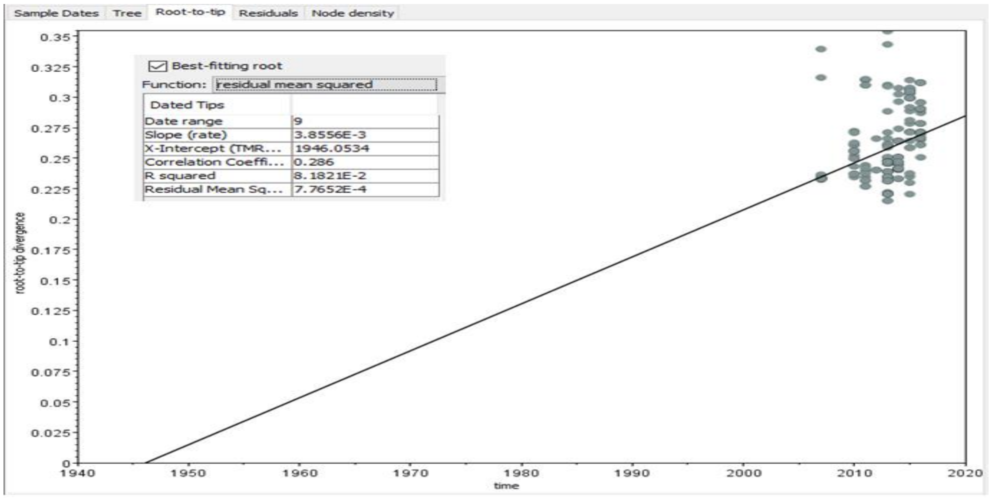

B

SupplimentaryFigure 1. Root to tip regression analysis plots of the genetic distances against the year of isolation for the Bt-CoV sequences analyzed in this study. Figure 2a represents Afr-Bt-CoV, while 2b represents sequences of African, Asian and European origins.
