# Supplementary material for: Evolutionary dynamics and geographic dispersal of beta coronaviruses in African bats
Source: PeerJ. 2020 Nov 26;8:e10434. doi: 10.7717/peerj.10434 (PMC7700737; doi:10.7717/peerj.10434)
Supplement: Supplemental Information 2 [file peerj-08-10434-s002.docx]

10 20 30 40 50 60 70 80 90 100

....|....|....|....|....|....|....|....|....|....|....|....|....|....|....|....|....|....|....|....|

**gb:KU131210 BatCoV/NIG/2011/13** **gtacttgttgtactagttcagagcggttttatagattagctaatgagtgtgcacaggtccttagtgagatggtcttgtgcggtggtgggttttatgtaaa**

**gb:KU131214 BatCoV/NGR/E.heivu** **gtacttgttgtactagttcagagcggttttatagattagctaatgagtgtgcacaggtccttagtgagatggtcttgtgcggtggtgggttttatgtaaa**

**gb:KU131215 BatCoV/NGR/E.heivu** **gtacttgttgtactagttcagagcggttttatagattagctaatgagtgtgcacaggtccttagtgagatggtcttgtgcggaggtgggttttatgtaaa**

**gb:KU131211 BatCoV/NGR/2011/13** **gtacttgttgtactagttcagagcgtttttatagattagctaatgagtgtgcacaggtccttagtgaaatggtcttgtgcggaggtgggttttatgtaaa**

**gb:KU131212 BatCoV/NGR/2011/13** **gtacttgttgtactagttcagagcgtttttatagattagctaatgagtgtgcacaggtccttagtgaaatggtcttgtgcggaggtgggttttatgtaaa**

**GU065432.1 BtCoV/KEN/E.heivum/** **gtacttgttgtactagttcagagcgtttttatagattagctaatgagtgtgcacaggtccttagtgagatggtcttgtgcggaggtgggttttatgtaaa**

**GU065431.1 BtCoV/KEN/E.heivum/** **gtacttgttgtactagttcagagcgtttttatagattagctaatgagtgtgcacaggtccttagtgagatggtcttgtgcggaggtgggttttatgtaaa**

**gb:KU131213 BatCoV/NGR/E.heivu** **gtacttgttgtactagttcagagcggttttatagattagctaatgagtgtgcacaggtccttagtgagatggtcttgtgcggaggtgggttttatgtaaa**

**GU065384.1 BtCoV/KEN/E. heivum** **gtacttgttgtactagttcagagcgtttttatagattagctaatgagtgtgcacaggtccttagtgagatggtcttgtgcggaggtgggttttatgtaaa**

**GU065442.1 BtCoV/KEN/E. heivum** **gtacttgttgtactagttcagagcgtttttatagattagctaatgagtgtgcacaggtccttagtgagatggtcttgtgcggaggtgggttttatgtaaa**

**GU065395.1 Bt CoV/KEN/E. heivu** **gtacttgttgtactagttcagagcgtttttatagattagctaatgagtgtgcacaggtccttagtgagatggtcttgtgcggaggtgggttttatgtaaa**

**GU065377.1 BtCoV/KEN/E.heivum/** **gtacttgttgtactagttcagagcgtttttatagattagctaatgagtgtgcacaggtccttagtgagatggtcttgtgcggaggtgggttttatgtaaa**

**GU065378.1 BtCoV/KEN/E.heivum/** **gtacctgttgtactagttcagagcggttttatagattagctaatgagtgtgcacaggtccttagtgagatggtcttgtgcggaggtgggttttatgtaaa**

**GU065437.1 BtCoV/KEN/H.heivum/** **gtacttgttgtactagttcagagcggttttatagattagctaatgagtgtgcacaggtccttagtgagatggtcttgtgcggaggtgggttttatgtaaa**

**gb:KX284999 /PREDICT-GVF-CM-EC** **gcacgtgttgtagtggttctgatcggttttatagacttgctaatgagtgtgcacaggtgcttagtgagatggtactttgtggtggtgggttttatgttaa**

**gb:KX285001 PREDICT-GVF-CM-ECO** **gcacgtgttgtagtggttctgatcggttttatagacttgctaatgagtgtgcacaggtgcttagtgagatggtactttgtggtggtgggttttatgttaa**

**gb:KX285008 PREDICT-GVF-CM-ECO** **gcacgtgttgtagtggttctgatcggttttatagacttgctaatgagtgtgcacaggtgcttagtgagatggtactttgtggtggtgggttttatgttaa**

**gb:KX285009 PREDICT-GVF-CM-ECO** **gcacgtgttgtagtggttctgatcggttttatagacttgctaatgagtgtgcacaggtgcttagtgagatggtactttgtggtggtgggttttatgttaa**

**gb:KX285012 PREDICT-GVF-CM-ECO** **gcacgtgttgtagtggttctgatcggttttatagacttgctaatgagtgtgcacaggtgcttagtgagatggtactttgtggtggtgggttttatgttaa**

**gb:KX285023 PREDICT-GVF-CM-ECO** **gcacgtgttgtagtggttctgatcggttttatagacttgctaatgagtgtgcacaggtgcttagtgagatggtactttgtggtggtgggttttatgttaa**

**gb:KX284994 PREDICT-GVF-CM-ECO** **gcacgtgttgtagtggttctgatcggttttatagacttgctaatgagtgtgcacaggtgcttagtgagatggtactttgtggtggtgggttttatgttaa**

**gb:KX284993 PREDICT-GVF-CM-ECO** **gcacgtgttgtagtggttctgatcggttttatagacttgctaatgagtgtgcacaggtgcttagtgagatggtactttgtggtggtgggttttatgttaa**

**gb:KX284951 PREDICT-GVF-CM-ECO** **gcacgtgttgtagtggttctgatcggttttatagacttgctaatgagtgtgcacaggtgcttagtgagatggtactttgtggtggtgggttttatgttaa**

**gb:KX284954 PREDICT-GVF-CM-ECO** **gcacgtgttgtagtggttctgatcggttttatagacttgctaatgagtgtgcacaggtgcttagtgagatggtactttgtggtggtgggttttatgttaa**

**gb:KX285006 PREDICT-GVF-CM-ECO** **gcacgtgttgtagtggttctgatcggttttatagacttgctaatgagtgtgcacaggtgcttagtgagatggtactttgtggtggtgggttttatgttaa**

**gb:KX285007 :PREDICT-GVF-CM-EC** **gcacgtgttgtagtggttctgatcggttttatagacttgctaatgagtgtgcacaggtgcttagtgagatggtactttgtggtggtgggttttatgttaa**

**gb:KX284989 PREDICT-GVF-CM-ECO** **gcacgtgttgtagtggttctgatcggttttatagacttgctaatgagtgtgcacaggtgcttagtgagatggtactttgtggtggtgggttttatgttaa**

**gb:KX284990 PREDICT-GVF-CM-ECO** **gcacgtgttgtagtggttctgatcggttttatagacttgctaatgagtgtgcacaggtgcttagtgagatggtactttgtggtggtgggttttatgttaa**

**gb:KX284986 PREDICT-GVF-CM-ECO** **gcacgtgttgtagtggttctgatcggttttatagacttgctaatgagtgtgcacaggtgcttagtgagatggtactttgtggtggtgggttttatgttaa**

**gb:KX284987 PREDICT-GVF-CM-ECO** **gcacgtgttgtagtggttctgatcggttttatagacttgctaatgagtgtgcacaggtgcttagtgagatggtactttgtggtggtgggttttatgttaa**

**gb:KX285075 PREDICT-CD115912/D** **gcacgtgttgtagtggttctgatcggttttatagacttgctaatgagtgtgcacaggtgcttagtgagatggtactttgtggtggtgggttttatgttaa**

**gb:KX285076 PREDICT-CD115914/d** **gcacgtgttgtagtggttctgatcggttttatagacttgctaatgagtgtgcacaggtgcttagtgagatggtactttgtggtggtgggttttatgttaa**

**gb:KX285024 /PREDICT-GVF-CM-EC** **gcacgtgttgtagtggttctgatcgtttttatagacttgctaatgagtgtgcacaggtgcttagtgagatggtactttgtggtggtgggttttatgttaa**

**gb:KX285025 PREDICT-GVF-CM-ECO** **gcacgtgttgtagtggttctgatcgtttttatagacttgctaatgagtgtgcacaggtgcttagtgagatggtactttgtggtggtgggttttatgttaa**

**gb:KX284957 PREDICT-GVF-CM-ECO** **gcacgtgttgtagtggttctgatcgtttttatagacttgctaatgagtgtgcacaggtgcttagtgagatggtactttgtggtggtgggttttatgttaa**

**gb:KX284958 PREDICT-GVF-CM-ECO** **gcacgtgttgtagtggttctgatcgtttttatagacttgctaatgagtgtgcacaggtgcttagtgagatggtactttgtggtggtgggttttatgttaa**

**gb:KX284985 PREDICT-GVF-CM-ECO** **gcacgtgttgtagtggttctgatcgtttttatagacttgctaatgagtgtgcacaggtgcttagtgagatggtactttgtggtggtgggttttatgttaa**

**gb:KX285427 PREDICT-130518Bt34** **gcacttgttgtagtggttctgatcggttttatagacttgctaatgaatgtgcacaggtgcttagtgagatggtactttgtggtggtgggttttatgttaa**

**gb:KX285428 PREDICT-130518Bt3/** **gcacttgttgtagtggttctgatcggttttatagacttgctaatgaatgtgcacaggtgcttagtgagatggtactttgtggtggtgggttttatgttaa**

**gb:KX285429 PREDICT-130518Bt35** **gcacttgttgtagtggttctgatcggttttatagacttgctaatgaatgtgcacaggtgcttagtgagatggtactttgtggtggtgggttttatgttaa**

**gb:KX285431 PREDICT-140403Bt16** **gcacttgttgtagtggttctgatcggttttatagacttgctaatgaatgtgcacaggtgcttagtgagatggtactttgtggtggtgggttttatgttaa**

**gb:KX285099 PREDICT-CD116096/D** **gcacttgttgtagtggttcggatcggttttatagacttgctaatgaatgtgcacaggtgcttagtgagatggtactttgtggtggtgggttttatgttaa**

**gb:KX285077 PREDICT-CD115937/D** **gcacgtgttgtagtggttctgatcggttttatagacttgctaatgaatgtgcacaggtgcttagtgagatggtactttgtggtggtgggttttatgttaa**

**gb:KX285078 PREDICT-CD115938/d** **gcacgtgttgtagtggttctgatcggttttatagacttgctaatgaatgtgcacaggtgcttagtgagatggtactttgtggtggtgggttttatgttaa**

**gb:KX285080 PREDICT-CD115941/D** **gcacgtgttgtagtggttctgatcggttttatagacttgctaatgaatgtgcacaggtgcttagtgagatggtactttgtggtggtgggttttatgttaa**

**gb:KX285082 PREDICT-CD115947/D** **gcacgtgttgtagtggttctgatcggttttatagacttgctaatgaatgtgcacaggtgcttagtgagatggtactttgtggtggtgggttttatgttaa**

**gb:KX285085 PREDICT-CD115956/D** **gcacgtgttgtagtggttctgatcggttttatagacttgctaatgaatgtgcacaggtgcttagtgagatggtactttgtggtggtgggttttatgttaa**

**gb:KX285087 PREDICT-CD116004/D** **gcacgtgttgtagtggttctgatcggttttatagacttgctaatgaatgtgcacaggtgcttagtgagatggtactttgtggcggtgggttttatgttaa**

**gb:KX285091 PREDICT-CD116015/D** **gcacgtgttgtagtggttctgatcggttttatagacttgctaatgaatgtgcacaggtgcttagtgagatggtactttgtggcggtgggttttatgttaa**

**gb:KX285088 PREDICT-CD116006/D** **gcacgtgttgtagtggttctgatcggttttatagacttgctaatgaatgtgcacaggtgcttagtgagatggtactttgtggcggtgggttttatgttaa**

**gb:KX285070 PREDICT-CD115124A/** **gcacgtgttgtagtggttctgatcgtttttatagacttgctaatgaatgtgcacaggtgcttagtgagatggtactttgtggtggtgggttttatgttaa**

**gb:KX285071 PREDICT-CD115222/D** **gcacgtgttgtagtggttctgatcgtttttatagacttgctaatgaatgtgcacaggtgcttagtgagatggtactttgtggtggtgggttttatgttaa**

**gb:KX285086 PREDICT-CD115975/D** **gcacgtgttgtagtggttctgatcgtttttatagacttgctaatgaatgtgcacaggtgcttagtgagatggtactttgtggtggtgggttttatgttaa**

**gb:KX285100 PREDICT-CD116101/D** **gcacgtgttgtagtggttctgatcgtttttatagacttgctaatgaatgtgcacaggtgcttagtgagatggtactttgtggtggtgggttttatgttaa**

**gb:KX285105 PREDICT-CD116107/D** **gcacgtgttgtagtggttctgatcgtttttatagacttgctaatgaatgtgcacaggtgcttagtgagatggtactttgtggtggtgggttttatgttaa**

**gb:KX285081 PREDICT-CD115943/D** **acacgtgttgtagtggttctgatcgtttttatagacttgctaatgaatgtgcacaggtgcttagtgagatggtactttgtggtggtgggttttatgttaa**

**gb:KX285101 PREDICT-CD116102/D** **gcacgtgttgtagtggttccgatcgtttttatagacttgctaatgaatgtgcacaggtgcttagtgagatggtactttgtggtggtgggttttatgttaa**

**gb:KX285102 PREDICT-CD116103/D** **gcacgtgttgtagtggttctgatcgtttttatagactcgctaatgaatgtgcacaggtgcttagtgagatggtactttgtggtggtgggttttatgttaa**

**gb:KX285103 PREDICT-CD116105/D** **gcacgtgtggtagtggttttgttcgtttttatagacttgataatgaatgtgcacaggtgcttagtgagatggtactttgtggtggtgggttttatgttaa**

**gb:MG762628 BtCoV/HKN/HKU9/Rou** **caacttgttgcaatgcttcggagcgattttataggttggctaatgagtgtgcccaggttttaagtgaaatggtcctttgtgggggcggtttttatgtgaa**

**gb:MG762630 BtCoV/HKN/HKU9/Rou** **caacttgttgcaatgcttcggagcgattttataggttggctaatgagtgtgcccaggttttaagtgaaatggtcctttgtgggggcggtttttatgtgaa**

**gb:MG762631 BtCoV/HKN/HKU9/Rou** **caacttgttgcaatgcttcggagcgattttataggttggctaatgagtgtgcccaggttttaagtgaaatggtcctttgtgggggcggtttttatgtgaa**

**gb:MG762633 BtCoV/HKN/HKU9/Rou** **caacttgttgcaatgcttcggagcgattttataggttggctaatgagtgtgcccaggttttaagtgaaatggtcctttgtgggggcggtttttatgtgaa**

**gb:MG762634 BtCoV/HKN/HKU9/Rou** **caacttgttgcaatgcttcggagcgattttataggttggctaatgagtgtgcccaggttttaagtgaaatggtcctttgtgggggcggtttttatgtgaa**

**gb:MG762635 BtCoV/HKN/HKU9/Rou** **caacttgttgcaatgcttcggagcgattttataggttggctaatgagtgtgcccaggttttaagtgaaatggtcctttgtgggggcggtttttatgtgaa**

**gb:MG762638 BtCoV/HKN/HKU9/Rou** **caacttgttgcaatgcttcggagcgattttataggttggctaatgagtgtgcccaggttttaagtgaaatggtcctttgtgggggcggtttttatgtgaa**

**gb:MG762639 BtCoV/HKN/HKU9/Rou** **caacttgttgcaatgcttcggagcgattttataggttggctaatgagtgtgcccaggttttaagtgaaatggtcctttgtgggggcggtttttatgtgaa**

**gb:MG762640 BtCoV/HKN/HKU9/Rou** **caacttgttgcaatgcttcggagcgattttataggttggctaatgagtgtgcccaggttttaagtgaaatggtcctttgtgggggcggtttttatgtgaa**

**gb:MG762641 BtCoV/HKN/HKU9/Rou** **caacttgttgcaatgcttcggagcgattttataggttggctaatgagtgtgcccaggttttaagtgaaatggtcctttgtgggggcggtttttatgtgaa**

**gb:MG762646 BtCoV/HKN/HKU9/Rou** **caacttgttgcaatgcttcggagcgattttataggttggctaatgagtgtgcccaggttttaagtgaaatggtcctttgtgggggcggtttttatgtgaa**

**gb:MG762650 BtCoV/HKN/HKU9/Rou** **caacttgttgcaatgcttcggagcgattttataggttggctaatgagtgtgcccaggttttaagtgaaatggtcctttgtgggggcggtttttatgtgaa**

**gb:MG762629 BtCoV/HKN/HKU9/Rou** **caacttgttgcaatgcttcggagcgattttataggttggctaatgagtgtgcccaggttttaagtgaaatggtcctttgtgggggcggtttttatgtgaa**

**gb:MG762643 BtCoV/HKN/HKU9/Rou** **caacttgttgcaatgcttcggagcgattttataggttggctaatgagtgtgcccaggttttaagtgaaatggtcctttgtgggggcggtttttatgtgaa**

**gb:MG762636 BtCoV/HKN/HKU9/Rou** **caacttgttgcaatgcttcggagcgattttataggttggctaatgagtgtgcccaggttttaagtgaaatggtcctttgtgggggcggtttttatgtgaa**

**gb:KX285051 BtCoV/MYS/PREDICT_** **ccacttgctgtaatgcctctgaaagattttataggctagcaaatgagtgtgctcaagtgctaagtgaaatggttttatgtggaggtggattctatgtaaa**

**gb:KX285112 Bt/CoV/Philippines** **ctacctgttgtaatgcttccgaaaggttttatcggttagcaaatgagtgtgcacaggtgcttagtgaaatggttctatgtggaggtggcttctatgtaaa**

**gb:KX285113 BtCoV/Philippines/** **ctacttgttgtaatgcttccgaaaggttttatcggttagcaaatgagtgtgcacaggtgcttagtgaaatggttctatgtggaggtggcttctatgtaaa**

**gb:KX285114 BtCoV/Philippines/** **ctacttgttgtaatgcttccgaaaggttttatcggttagcaaatgagtgtgcacaggtgcttagtgaaatggttctatgtggaggtggcttctatgtaaa**

**KP696742.1 BtCoV/MDG/ANK036F/2** **agacttgttgtaactcatcagaacgattttatcgtcttgctaatgagtgtgcacaagttttgtgtgaaatggtattgtgcggtggcgggttttatgttaa**

**KP696744.1 BtCoV/MDG/ BEM073F/** **agacttgttgtaactcatcagaacgattttatcgtcttgctaatgagtgtgcacaagttttgtgtgaaatggtattgtgcggtggcgggttttatgttaa**

**KP696747.1 BtCoV/MDG/ANK081F/2** **agacttgttgtaactcatcagaacgattttatcgtcttgctaatgagtgtgcacaagttttgtgtgaaatggtattgtgcggtggcgggttttatgttaa**

**KP696746.1 BtCoV/MDG/BEM077F/2** **agacttgttgtaatttctcagagcggttttatagacttgctaatgagtgtgcacaagttctttgtgaaatggtgttatgtggtggcgggttttatgttaa**

**KP696745.1 BtCoV/MDG/BEM074F/2** **agacttgttgtaatttctcagagcggttttatagacttgctaatgagtgtgcacaagttctttgtgaaatggtgttatgtggtggcgggttttatgttaa**

**gb:KT717381 BatCoV Art.lit/206** **gtggttgttgtaacggtaatgagcgtttttaccgtttagcaaatgagtgtgcacaagtgcttagtgagatggttctatgcactggtgggttttatgtaaa**

**gb:KX285064 BtCoV PREDICT_CoV-** **ctgggtgttgtaacagactggaacggttttaccgactagccaatgagtgtgcccaggtgttaagtgagatagttttgtgtacaggtggcttctatgttaa**

**gb:MG193617 BtCoV/20161014_DC1** **gcacttgttgtactacaagagacagattctatcgcttagcaaatgagtgcgctcaagtgctaagtgaatatgttctatgcggtggtggttactacgttaa**

**gb:MG252876 BtCoV/20161014_DC1** **gcacttgttgtactacaagagacagattctatcgcttagcaaatgagtgcgctcaagtgctaagtgaatatgttctatgcggtggtggttactacgttaa**

**gb:MG310223 BtCoV/20161014_DC_** **gcacttgttgtactacaagagacagattctatcgcttagcaaatgagtgcgctcaagtgctaagtgaatatgttctatgcggtggtggttactacgttaa**

**gb:MG310233 BtCoV/20161011_DC1** **gcacttgttgtactacaagagacagattctatcgcttagcaaatgagtgcgctcaagtgctaagtgaatatgttctatgcggtggtggttactacgttaa**

**gb:MG310243 BtCoV/20150816HFP_** **gcacttgttgtactacaagagacagattctatcgcttagcaaatgagtgcgctcaagtgctaagtgaatatgttctgtgcggtggtggttactacgttaa**

**gb:MG205593 BtCoV/20150720ABA_** **gcacttgttgtactacaagggacagattttatcgcttagcaaatgagtgtgctcaagtgctaagtgaatatgttttatgcggtggtggttactacgttaa**

**gb:MG310245 BtCoV/20150720ABA_** **gcacttgttgtactacaagggacagattttatcgcttagcaaatgagtgtgctcaagtgctaagtgaatatgttttatgcggtggtggttactacgttaa**

**gb:MG817483 BtCoV/20140127ABA_** **gcacttgttgtactacaagggacagattttatcgcttagcaaatgagtgtgctcaagtgctaagtgaatatgttttatgcggtggtggttactacgttaa**

**gb:MG310225 BtCoV/20141022HBI_** **gcacttgttgtactacaagggacagattttatcgcttagcaaatgagtgtgctcaagtgttaagtgaatatgttctatgcggtggtggttattacgttaa**

**gb:MG205595 BtCoV/20150816HFP_** **gcacttgttgtactacaagagacagattttatcgcttagcaaatgagtgcgctcaagtgctaagtgaatatgttctatgcggtggtggttactacgttaa**

**gb:MG205596 BtCoV/20150816HFP_** **gcacttgttgtactacaagagacagattttatcgcttagcaaatgagtgcgctcaagtgctaagtgaatatgttctatgcggtggtggttactatgttaa**

**gb:MG205597 BtCoV/20150816HFP_** **gcacttgttgtactacaagagacagattttatcgcttagcaaatgagtgcgctcaagtgctaagtgaatatgttctatgcggtggtggttactatgttaa**

**gb:MG310229 BtCoV/20150819LFU_** **gcacttgttgtactacaagagacagattttatcgcttagcaaatgagtgcgctcaagtgctaagtgaatatgttytatgcggtggtggttactatgttaa**

**gb:MG817484 BtCoV/20141103SRP** **gcacttgttgtactacaagagacagattttatcgcttagcaaatgagtgcgctcaagtgctaagtgaatatgttttatgtggtggtggttattacgttaa**

**gb:MG252869 BtCoV/20160303FEK_** **gcacttgttgtactacaagagacagattttatcgcttagcaaatgagtgtgctcaagtgctaagtgaatatgttttatgtggtggtggttactacgttaa**

**gb:MG252872 BtCoV/20160304FEK** **gcacttgttgtactacaagagacagattttatcgcttagcaaatgagtgtgctcaagtgctaagtgaatatgttttatgtggtggtggttactacgttaa**

**gb:MG252873 BtCoV/20160304FEK_** **gcacttgttgtactacaagagacagattttatcgcttagcaaatgagtgtgctcaagtgctaagtgaatatgttttatgtggtggtggttactacgttaa**

**gb:MG310226 BtCoV/20150105CGR_** **gcacttgttgtactacaagagacagattttatcgcttagcaaatgagtgtgctcaagtgctaagtgaatatgttttatgtggtggtggttactacgttaa**

**gb:MG817485 BtCoV/20150105CGR_** **gcacttgttgtactacaagagacagattttatcgcttagcaaatgagtgtgctcaagtgctaagtgaatatgttttatgtggtggtggttactacgttaa**

**gb:MG817486 BtCoV/20150105CGR_** **gcacttgttgtactacaagagacagattttatcgcttagcaaatgagtgtgctcaagtgctaagtgaatatgttttatgtggtggtggttactacgttaa**

**gb:MG205591 BtCoV/20150106CDK_** **gcacttgttgtactacaagagacaggttttaccgtttagcaaatgagtgtgctcaagtgctaagcgaatatgttttatgtggtggtggttattacgttaa**

**gb:MG252862 BtCoV/20150919CDK_** **gcacttgttgtactacaagagacaggttttaccgtttagcaaatgagtgtgctcaagtgctaagcgaatatgttttatgtggtggtggttattacgttaa**

**gb:MG817496 BtCoV/20150919CDK2** **gcacttgttgtactacaagagacaggttttaccgtttagcaaatgagtgtgctcaagtgctaagcgaatatgttttatgtggtggtggttattacgttaa**

**gb:MG252859 BtCoV/20150920CGC_** **gcacttgttgtactacaagagacaggttttaccgtttagcaaatgagtgtgctcaagtgctaagcgaatatgttctatgtggtggtggttattacgttaa**

**gb:MG817488 BtCoV/20150107CGC_** **gcacttgttgtactacaagagacaggttttaccgtttagcaaatgagtgtgctcaagtgctaagcgaatatgttctatgtggtggtggttattacgttaa**

**gb:MG817494 BtCoV/20150920CGC_** **gcacttgttgtactacaagagacaggttttaccgtttagcaaatgagtgtgctcaagtgctaagcgaatatgttctatgtggtggtggttattacgttaa**

**gb:MG252864 BtCoV/20150108CCK_** **gcacttgttgtactacaagagacagattttaccgcttagcaaatgagtgtgctcaagtgctaagcgaatatgttttatgtggtggtggttattacgttaa**

**KC776174.1 Human MERS/CoV 2c J** **gcacttgttgtactacaagggacagattttatcgcttggcaaatgagtgtgctcaggtgctaagcgaatatgttctatgtggtggtggttactacgtcaa**

**MG923474.1 MERS-CoV camel/Nige** **gcacttgttgtactacaagggacagattttatcgcttggcaaatgagtgtgctcaggtgctaagcgaatatgttctatgtggtggtggttactacgtcaa**

**gb:KF500943 BtCoV/Pipistrellus** **atacttgttgtacaacaagagacagattttaccgtttagcaaatgagtgcgctcaggtgttaagtgagtatgtgttatgcggtggtggttattacgttaa**

**gb:KF500944 BtCoV/Pipistrellus** **atacttgttgtacaacaagagacagattttaccgtttagcaaatgagtgcgctcaggtgttaagtgagtatgtgttatgcggtggtggttattacgttaa**

**gb:KF500942 BtCoV/Pipistrellus** **atacttgttgtacaacaagagatagattttaccgtttagcaaatgagtgcgctcaggtgttaagtgagtatgtgttatgcggtggtggttattacgttaa**

**gb:KF500946 BtCoV/Pipistrellus** **atacttgttgtacaacaagagatagattttaccgtttagcaaatgagtgtgctcaggtgttaagtgaatatgtgttatgcggtggtggttattacgttaa**

**gb:KF500947 BtCoV/Pipistrellus** **atacttgttgtacaacaagagatagattttaccgtttagcaaatgagtgtgctcaggtgttaagtgaatatgtgttatgcggtggtggttattacgttaa**

**gb:HQ184059 BatCoV/H.sav/J/Spa** **atacttgttgtacaacaagagatagattttaccgcttagcaaatgagtgtgctcaagtgctaagtgaatatgttttatgtggtggtggttattacgttaa**

**gb:KT717386 BatCoV Eum.gla/242** **acacttgttgcagtacaagtgatagattttaccgcttagctaatgagtgtgctcaagtgttaagtgaatatgtgttgtgtggtggtggttactacgtaaa**

**gb:GQ259977 BtCoV P.pipi/VM314** **gaacttgttgtactacaagggacagattttatcgcttggcaaatgagtgtgctcaggtgctgagtgaatatgtgttatgcggtggtggctactacgtcaa**

**gb:KX285197 BtCoV/CHN/HKU5/PRE** **gtacttgttgtactaatactgatagattttaccgcttagcaaatgagtgcgctcaagtgcttagtgagtatgtcctttgtggcggtggttattacgtcaa**

**gb:KX285199 BtCoV/CHN/HKU5/PRE** **gtacatgttgtactaatactgatagattttaccgcttagcaaatgagtgcgctcaagtgcttagtgagtatgtcctttgtggtggtggttattacgtcaa**

**gb:KX285200 BtCoV/CHN/HKU5/PRE** **gtacttgttgtactaatactgatagattctaccgcttagcaaatgagtgcgctcaagtgcttagcgagtatgtcctctgtggtggtggttattacgtcaa**

**gb:HQ184062 BtCoV/E.isa/M/Spai** **gtacttgctgctctaccagtgatcggttctaccgcttagctaatgagtgcgctcaagtgctaagtgaatatgtgttatgcggtggtggttactatgttaa**

**gb:KC522038 BtCoV/HKN/Tylonyct** **gtacttgttgtactaattctgataggttctaccgcttagccaatgagtgtgctcaagtgttaagtgagtatgtcctatgcggagggggttattatgttaa**

**gb:KC522039 BtCoV/HKN/Tylonyct** **gtacttgttgtactaattctgataggttctaccgcttagccaatgagtgtgctcaagtgttaagtgagtatgtcctatgcggagggggttattatgttaa**

**gb:KC522047 BtCoV/HKN/Tylonyct** **gtacttgttgtactaattctgataggttctaccgcttagccaatgagtgtgctcaagtgttaagtgagtatgtcctatgcggagggggttattatgttaa**

**gb:KC522042 BtCoV/HKN/Tylonyct** **gtacttgttgtactaattctgataggttctaccgcttagccaatgagtgtgctcaagtgttaagtgagtatgtcctatgcggagggggttattatgttaa**

**gb:KC522043 BtCoV/HKN/Tylonyct** **gtacttgttgtactaattctgataggttctaccgcttagccaatgagtgtgctcaagtgttaagtgagtatgtcctatgcggagggggttattatgttaa**

**gb:KC522036 BtCoV/HKN/Tylonyct** **gtacttgttgtactaattctgataggttctaccgcttagccaatgagtgtgctcaagtgttaagtgagtatgtcctatgcggaggaggttattatgttaa**

**gb:KC522037 BtCoV/HKN/Tylonyct** **gtacttgttgtactaattctgataggttctaccgcttagccaatgagtgtgctcaagtgttaagtgagtatgtcctatgcggaggtggttattatgttaa**

**gb:KX447563 BatSARS/CHN/HKU3/1** **gcacttgttgtaacttgtcacatcgtttctatagattagctaatgagtgtgcacaagtattaagtgagatggtcatgtgtggcggctcattatatgtgaa**

**gb:KX447564 BatSARS/CHNHKU3/15** **gcacttgttgtaacttgtcacatcgtttctatagattagctaatgagtgtgcacaagtattaagtgagatggtcatgtgtggcggctcattatatgtgaa**

**gb:MG772855 BtCoV/CHN/SARS-lik** **gcacttgttgtaacttgtcacaccgtttctatagattagctaatgagtgtgcacaagtattaagtgagatggtcatgtgtggcggctcattatatgtgaa**

**gb:MG772880 BtCoV/CHN/SARS-lik** **gcacttgttgtaacttgtcacaccgtttctatagattagctaatgagtgtgcacaagtattaagtgagatggtcatgtgtggcggctcattatatgtgaa**

**gb:MG772855 SARS-like bat-SL-C** **gcacttgttgtaacttgtcacaccgtttctatagattagctaatgagtgtgcacaagtattaagtgagatggtcatgtgtggcggctcattatatgtgaa**

**gb:MG772883 BtCoV/CHN/SARS-lik** **gcacttgttgtaacttgtcacaccgtttctatagattagctaatgagtgtgcacaagtattaagtgagatggtcatgtgtggcggctcattatatgtgaa**

**gb:MG772862 BtCoV/CHNSARS-like** **gcacttgttgtaacttgtcacaccgtttctatagattagctaatgagtgtgcacaagtattaagtgagatggtcatgtgtggcggctcattatatgtgaa**

**gb:MG772879 SARS-like bat-SL-C** **gcacttgttgtaacttgtcacaccgtttctatagattagctaatgagtgtgcacaagtattaagtgagatggtcatgtgtggcggctcattatatgtgaa**

**gb:MG772858 BtCoV/CHN/SARS-lik** **gcacttgttgtaacttgtcacaccgtttctatagattagctaatgagtgtgcacaaatattaagtgagatggtcatgtgtggcggctcattatatgtgaa**

**gb:MG772859 SARS-like bat-SL-C** **gcacttgttgtaacttgtcacaccgtttctatagattagctaatgagtgtgcacaaatattaagtgagatggtcatgtgtggcggctcattatatgtgaa**

**gb:MG772858 SARS-like bat-SL-C** **gcacttgttgtaacttgtcacaccgtttctatagattagctaatgagtgtgcacaaatattaagtgagatggtcatgtgtggcggctcattatatgtgaa**

**gb:KX285125 BatSARS/CHN/HKU3/P** **gcacttgttgtaacttgtcacaccgtttctatagattagctaatgagtgtgcacaagtattaagtgagatggtcatgtgtggcggctcattatatgtgaa**

**gb:KX285125 BtCoV/SARS/CHN/HKU** **gcacttgttgtaacttgtcacaccgtttctatagattagctaatgagtgtgcacaagtattaagtgagatggtcatgtgtggcggctcattatatgtgaa**

**gb:MG772861 SARS-like bat-SL-C** **gcacttgttgtaacttgtcacaccgtttttatagattagctaatgagtgtgcacaagtattaagtgagatggtcatgtgtggcggctcattatatgtgaa**

**gb:MG772852 BtCoV/CHN/SARS-lik** **gcacttgttgtaacttgtcacaccgtttttatagattagctaatgagtgtgcacaaatattaagtgagatggtcatgtgtggcggctcattatatgtgaa**

**gb:MG772875 BtCoV/CHNSARS-like** **gcacttgttgtaacttgtcacaccgtttttatagattagctaatgagtgtacacaagtattaagtgagatggtcatgtgtggcggctcattatatgtgaa**

**gb:MG772874 BtCoV/CHN/SARS-lik** **gcacttgttgtaacttgtcacaccgtttttatagattagctaatgagtgtacacaagtattaagtgagatggtcatgtgtggcggctcattatatgtgaa**

**gb:MG772865 BtCoV/CHN/SARS-lik** **gcacttgttgtaacttgtcacaccgtttttatagattagctaatgagtgtacacaagtattaagtgagatggtcatgtgtggcggctcattatatgtgaa**

**gb:MG772863 BtCoV/CHN/SARS-lik** **gcacttgttgtaacttgtcacaccgtttttatagattagctaatgagtgtacacaagtattaagtgagatggtcatgtgtggcggctcattatatgtgaa**

**gb:MG772891 BtCoV/CHN/SARS-lik** **gcacttgttgtaacttgtcacaccgtttttatagattagctaatgagtgtacacaagtattaagagagatggtcatgtgtggcggctcattatatgtgaa**

**gb:MG772891 SARS-like bat-SL-C** **gcacttgttgtaacttgtcacaccgtttttatagattagctaatgagtgtacacaagtattaagagagatggtcatgtgtggcggctcattatatgtgaa**

**gb:MG772890 BtCoV/CHN/SARS-lik** **gcacttgttgtaacttgtcacaccgtttttatagattagctaatgagtgtacacaagtattaagtgagatggtcatgtgtggcggctcattatatgtgaa**

**gb:MG772890 Bat SARS-like bat-** **gcacttgttgtaacttgtcacaccgtttttatagattagctaatgagtgtacacaagtattaagtgagatggtcatgtgtggcggctcattatatgtgaa**

**gb:MG772854 BtCoV/CHN/SARS-lik** **gcacttgttgtaacttgtcacaccgtttttatagattagctaatgagtgtgcacaagtattaagtgagatggtcatgtgtggcggctcattatatgtgaa**

**gb:MG772854 SARS-like bat-SL-C** **gcacttgttgtaacttgtcacaccgtttttatagattagctaatgagtgtgcacaagtattaagtgagatggtcatgtgtggcggctcattatatgtgaa**

**gb:MG772849 SARS-like bat-SL-C** **gcacttgttgtaacttgtcacaccgtttttatagattagctaatgagtgtgcacaagtattaagtgagatggtcatgtgtggcggctcattatatgtgaa**

**gb:MG772848 SARS-like bat-SL-C** **gcacttgttgtaacttgtcacaccgtttttatagattagctaatgagtgtgcacaagtattaagtgagatggtcatgtgtggcggctcattatatgtgaa**

**gb:MG772884 BtCoV/CHN/SARS-lik** **gcacttgttgtaacttgtcacaccgtttttatagattagctaatgagtgtacacaagtattaagtgagatggtcatgtgtggcggctcattatatgtgaa**

**gb:KY423420 BtCoV/FRA_EPI1_387** **gtacttgttgtaacctttcacaacgtttctacaggttagctaatgagtgtgctcaggtacttagtgaaatggtcatgtgtggcggttcactttatgtaaa**

**gb:KY423421 BtCoVFRA_EPI1_3871** **gtacttgttgtaacctttcacaacgtttctacaggttagctaatgagtgtgctcaggtacttagtgaaatggtcatgtgtggcggttcactttatgtaaa**

**gb:KY423431 BtCoV/FRA_EPI1_387** **gtacttgttgtaacctttcacaacgtttctacaggttagctaatgagtgtgctcaggtacttagtgaaatggtcatgtgtggcggttcactttatgtaaa**

**gb:KY423422 BtCoV/FRA_EPI1_387** **gtacttgttgtaacctttcacaacgtttctacaggttagctaatgagtgtgctcaggtacttagtgaaatggtcatgtgtggcggttcactttatgtaaa**

**gb:KY423428 BtCoV/FRA_EPI1_388** **gtacttgttgtaacctttcacaacgtttctacaggttagctaatgagtgtgctcaggtacttagtgaaatggtcatgtgtggcggttcactttatgtaaa**

**gb:KY423425 BtCoV/FRA_EPI1_388** **gtacttgttgtaacctttcacaacgtttctacaggttagctaatgagtgtgctcaggtacttagtgaaatggtcatgtgtggcggttcactttatgtaaa**

**gb:KY423430 BtCoV/FRA_EPI1_389** **gtacttgttgtaacctttcacaacgtttctacaggttagctaatgagtgtgctcaggtacttagtgaaatggtcatgtgtggcggttcactttatgtaaa**

**gb:KY423424 BtCoV/FRA_EPI1_389** **gtacttgttgtaacctttcacaacgtttctacaggttagctaatgagtgtgctcaggtacttagtgaaatggtcatgtgtggcggttcactttatgtaaa**

**gb:KY423432 BtCoV/FRA_EPI1_390** **gtacttgttgtaacctttcacaacgtttctacaggttagctaatgagtgtgctcaggtacttagtgaaatggtcatgtgtggcggttcactttatgtaaa**

**gb:KY423419 BtCoV/FRA_EPI1_394** **gtacttgttgtaacctttcacaacgtttctacaggttagctaatgagtgtgctcaggtacttagtgaaatggtcatgtgtggcggttcactttatgtaaa**

**gb:KY423427 BtCoV/FRA_EPI1_400** **gtacttgttgtaacctttcacaacgtttctacaggttagctaatgagtgtgctcaggtacttagtgaaatggtcatgtgtggcggttcactttatgtaaa**

**gb:KY423417 BtCoV/FRA_EPI1_401** **gtacttgttgtaacctttcacaacgtttctacaggttagctaatgagtgtgctcaggtacttagtgaaatggtcatgtgtggcggttcactttatgtaaa**

**gb:KY423397 FRA_EPI1_3875_3E_P** **gtacttgttgtaacctttcacaccgtttctacaggttagctaatgagtgtgctcaggtacttagtgaaatggtcatgtgtggcggttcactctatgtaaa**

**gb:KY423398 BtCoV/FRA_EPI1_388** **gtacttgttgtaacctttcacaccgtttctacaggttagctaatgagtgtgctcaggtacttagtgaaatggtcatgtgtggcggttcactctatgtaaa**

**gb:KY423399 BtCoV/FRA_EPI1_393** **gtacttgttgtaacctttcacaccgtttctacaggttagctaatgagtgtgctcaggtacttagtgaaatggtcatgtgtggcggttcactctatgtaaa**

**gb:KY423411 BtCoV/FRA_EPI1_394** **gtacttgttgtaacctttcacaccgtttctacaggttagctaatgagtgtgctcaggtacttagtgaaatggtcatgtgtggcggttcactctatgtaaa**

**gb:KY423400 BtCoV/FRA_EPI1_396** **gtacttgttgtaacctttcacaccgtttctacaggttagctaatgagtgtgctcaggtacttagtgaaatggtcatgtgtggcggttcactctatgtaaa**

**gb:KY423396 BtCoV/FRA_EPI1_400** **gtacttgttgtaacctttcacaccgtttctacaggttagctaatgagtgtgctcaggtacttagtgaaatggtcatgtgtggcggttcactctatgtaaa**

**gb:KY423395 BtCoV/FRA_EPI1_401** **gtacttgttgtaacctttcacaccgtttctacaggttagctaatgagtgtgctcaggtacttagtgaaatggtcatgtgtggcggttcactctatgtaaa**

**gb:KY502395 SARS-rel/BtCoV/Rhi** **gtacttgttgtaacctttcacaccgtttctacaggttagctaatgagtgtgctcaggtacttagtgaaatggtcatgtgtggcggttcactctatgtaaa**

**gb:KY423402 BtCoV/FRA_EPI1_387** **gtacttgttgtaacctttcacaccgtttctacaggttagctaatgagtgtgctcaggtacttagtgaaatggtcatgtgtggcggttcactttatgtaaa**

**gb:KY423403 BtCoV/FRA_EPI1_Rhf** **gtacttgttgtaacctttcacaccgtttctacaggttagctaatgagtgtgctcaggtgcttagtgaaatggtcatgtgtggcggttcactctatgtaaa**

**gb:KY423388 BtCoV/FRA_EPI1_Rhf** **gtacttgttgtaacctttcacaccgtttctacaggttagctaatgagtgtgctcaggtgcttagtgaaatggtcatgtgtggcggttcactctatgtaaa**

**gb:KY423389 BtCoV/FRA_EPI1_Rhf** **gtacttgttgtaacctttcacaccgtttctacaggttagctaatgagtgtgctcaggtgcttagtgaaatggtcatgtgtggcggttcactctatgtaaa**

**gb:KY423390 BtCoV/FRA_EPI1_Rhf** **gtacttgttgtaacctttcacaccgtttctacaggttagctaatgagtgtgctcaggtgcttagtgaaatggtcatgtgtggcggttcactctatgtaaa**

**gb:KY423394 BtCoV/FRA_EPI1_Rhf** **gtacttgttgtaacctttcacaccgtttctacaggttagctaatgagtgtgctcaggtgcttagtgaaatggtcatgtgtggcggttcactctatgtaaa**

**gb:KY423392 BtCoV/FRA_EPI1_Rhf** **gtacttgttgtaacctttcacaccgtttctacaggttagctaatgagtgtgctcaggtgcttagtgaaatggtcatgtgtggcggttcactctatgtaaa**

**gb:KY423391 BtCoV/FRA_EPI1_Rhf** **gtacttgttgtaacctttcacaccgtttctacaggttagctaatgagtgtgctcaggtgcttagtgaaatggtcatgtgtggcggttcactctatgtaaa**

**gb:KY423386 BtCoV/FRA_EPI1_Rhf** **gtacttgttgtaacctttcacaccgtttctacaggttagctaatgagtgtgctcaggtgcttagtgaaatggtcatgtgtggcggttcactctatgtaaa**

**gb:KY423412 BtCoV/SPA_EPI1_Rhf** **gtacttgttgtaacctttcacaccgtttctacaggttagctaatgagtgtgctcaggtacttagtgaaatggtcatgtgtggcggttcactctatgtaaa**

**gb:KY423375 BtCoV/FRA_EPI1_Rhf** **gtacttgttgtaacctttcacaccgtttctacaggttagctaatgagtgtgctcaggtgcttagtgaaatggtcatgtgtggcggttcactctatgtaaa**

**gb:KY423413 BtCoV/SPA_EPI1_Rhf** **gtacttgttgtaacctttcacaccgtttctacaggttagctaatgagtgtgctcaggtacttagtgaaatggtcatgtgtggcggttcactctatgtaaa**

**gb:KY423433 BtCoV/FRA_EPI1_396** **gtacttgttgtaacctttcacaccgtttctacaggttagctaatgagtgtgctcaggtacttagtgaaatggtcatgtgtggcggttcactctatgtaaa**

**gb:KY423437 BtCoV/FRA_EPI1_397** **gtacttgttgtaacctttcacaccgtttctacaggttagctaatgagtgtgatcaggtacttagtgaaatggtcatgtgtggcggttcactctatgtaaa**

**gb:KY423438 BtCoV/FRA_EPI1_396** **gtacttgttgtaacctttcacaacgtttctacaggttagctaatgagtgtgctcaggtacttagtgaaatggtcatgtgtggcggttcactttatgtaaa**

**gb:KC633214 BtCoV/Rhi_hip/R13-** **gtacttgttgtaacctttcacaccgcttctacaggttagctaacgagtgtgcacaggtacttagtgaaatggtcatgtgtggcggttcactctatgttaa**

**gb:KC633213 BtCoV/Rhi_hip/R46-** **gtacttgttgtaacctttcacaccgcttctacaggttagctaacgagtgtgcacaggtacttagtgaaatggtcatgtgtggcggttcactctatgttaa**

**gb:KC633212 BtCoV/Rhi_hip/R7-0** **gtacttgttgtaacctttcacaccgcttctacaggttagctaacgagtgtgcacaggtactcagtgaaatggtcatgtgtggcggttcactctatgttaa**

**gb:KC633209 BtCoV/Rhi_hip/R8-0** **gtacttgttgtaacctttcacaccgcttctacaggttagctaacgagtgtgcacaggtactcagtgaaatggtcatgtgtggcggttcactctatgttaa**

**gb:KC633210 BtCoV/Rhi_hip/R77-** **gtacctgttgtaacctttcacaccgcttctacaggttagctaacgagtgtgcacaggtactcagtgaaatggtcatgtgtggcggttcactctatgttaa**

**FJ710047.1 BtCoV/Ghana Kwam/20** **cgacgtgttgtacgctgagtgagcgatattatcgccttgccaatgaatgcgctcaagttttgagtgaaatggtactttgtggtggtgcactttatgtaaa**

**FJ710054.1 BtCoV Hipposideros/** **cgacgtgttgtacgctgagtgagcgatattatcgccttgccaatgaatgcgctcaagttttgagtgaaatggtactttgtggtggtgcactttatgtaaa**

**gb:HQ166910 ZBCoV**  **ctacatgctgtaatttgagtgaacggttttaccgacttgctaatgagtgcgctcaagttttgagcgaaatggttctgtgcggcggtgcgctttacgtaaa**

**gb:EU834950 BtCoV R.aur/Austra** **atacgtgctgtactgtgcgagataggttttaccgcttagctaatgagtgtgctcaagtgctgagcgagatggtgctatgtggtggttcgttttatgttaa**

**KP112152.1 HuCoV HKU1/HCOV/KEN** **aattttgttgttcacatggtgatagattttatcgccttgcgaatgaatgtgctcaagttttgagtgaaatagttatgtgtggcggttgctattatgttaa**

**KP112168.1 HuCoV/OC43 HCOV/KEN** **agacatgttgttcgcaaagcgataggttttatcgccttgcgaatgaatgcgcacaagttttgagtgaaattgttatgtgtggtggctgttattatgttaa**

**KP112167.1 UNVERIFIED: HuCoV/O** **agacatgttgttcgcaaagcgataggttttatcgccttgcgaatgaatgcgcacaagttttgagtgaaattgttatgtgtggtggctgttattatgttaa**

**KP112165.1 HuCoV/OC43 HCOV/KEN** **agacatgttgttcgcaaagcgataggttttatcgccttgcgaatgaatgcgcacaagttttgagtgaaattgttatgtgtggtggctgttattatgttaa**

**KP112163.1 HuCoV OC43 HCOV/KEN** **agacatgttgttcgcaaagcgataggttttatcgacttgcgaatgaatgcgcacaagttttgagtgaaattgttatgtgtggtggctgttattatgttaa**

**KP112161.1 HuCoV OC43 HCOV/KEN** **aaacctgctgcagccagagcgatcgtttttatcgtctggcgaacgaatgcgcgcaggtgctgagcgaaattgtgatgtgcggcggctgctattatgtgaa**

**KC886322.1 BatCoV P.davyi49/Me** **ttggctgctgtaaccactctgagaggttttacagacttgctaatgagtgtgcccaagtgctaagtgaggtggttctttgcactggcgggttttatgttaa**

**MG310257.1 alpha BtCoV/2014011** **ttaactgttgtacagcctctgaccgttattatcgactttgtaatgaacttgcccaagttctcactgaggttgtttattctaacggtggcttttattttaa**

**MG817498.1 alpha BtCoV/2015010** **ttaactgttgcacagcctcagaccgttattatcgactttgtaatgaacttgctcaagttcttactgaggttgtttattctaacggtggcttttatttcaa**

**MG310246.1 alpha BtCoV/2015010** **ttaactgttgcacagcctcagaccgttattatcgactttgtaatgaacttgctcaagttcttactgaggttgtttattctaacggtggcttttatttcaa**

**MG844332.1 alpha BtCoV/2014092** **ttaactgttgcacatcttctgaccgttattatcggctttgtaatgaacttgcgcaagttcttactgaagttgtttactctaacggcggtttttattttaa**

**gb:GQ259961 BtCoV N.noc/VM176/** **ttaattgttgtacttcctctgacagatattatcgtctctgtaatgaacttgctcaagttcttactgaggttgtttattctaatggaggtttttacttcaa**

**gb:GQ259968 BtCoV M.das/VM105/** **ttacatgctgtaactctagtgatagattttataggttgtgtaacgaattagcccaagtgctaactgaggtcgtttattctaatggtggtttctacctcaa**

**gb:GQ259969 BtCoV M.das/VM62/N** **ttacatgctgtaactctagtgatagattttacaggttgtgtaacgaattagcccaagtgctaactgaggtcgtctattctaatggtggtttctacctcaa**

**gb:GQ259970 BtCoV M.das/VM73/N** **ttacatgctgtaactctagtgatagattttacaggttgtgtaacgaattagcccaagtgctaactgaggtcgtctattctaatggtggtttctacctcaa**

**gb:GQ259965 BtCoV M.das/VM3/NL** **ttacatgctgtaactctagtgatagattttacaggttgtgtaacgagttagcccaagtgctaactgaggtcgtctattctaatggtggtttctacctcaa**

**gb:GQ259966 BtCoV M.das/VM34/N** **ttacatgctgtaactctagtgatagattttacaggttgtgtaacgagttagcccaagtgctaactgaggtcgtctattctaatggtggtttctacctcaa**

**gb:GQ259971 BtCoV M.dau/VM222/** **ttacatgctgtaattccattgataggttttacagactttgtaatgagctggcacaagtactaactgaggtcatctattctaatggtggtttctacctcaa**

**gb:GQ259973 BtCoV M.dau/VM361/** **ttacatgctgtaattccactgataggttttacagactttgtaatgagctggcacaagtactaactgaggtcatctattctaatggtggtttctacctcaa**

**MK603153.1 alpha BtCoV RIBSP-K** **ctacttgttgtagcaatactgatcgttattacagactttgcaatgagcttgcacaggttcttacagaggttgtttattccaatggtggcttctatatgaa**

**MK603159.1 alpha BtCoV RIBSP-K** **ctacttgttgtagcaatactgatcgttattacagactttgcaatgagcttgcacaggttcttacagaggttgtttattccaatggtggcttctatatgaa**

**MK603152.1 alpha CoV RIBSP-KZ-** **ctacttgttgtagcaatactgatcgttattacagactttgcaatgagcttgcacaggttcttacagaggttgtttattccaatggtggcttctatatgaa**

**MK603160.1 alpha RIBSP-KZ-BatC** **ctacttgttgtagcaatactgatcgttattacagactttgcaatgagcttgcacaggttcttacagaggttgtttattccaatggtggcttctatatgaa**

**MK603156.1 BtCoV alpha RIBSP-K** **ctacttgttgtagcaatactgatcgttattacagactttgcaatgagcttgcacaggttcttacagaggttgtttattccaatggtggcttctatatgaa**

**MK603157.1 BtCoV alpha RIBSP-K** **caacttgttgtagcaatactgatcgttattatagactttgcaatgagcttgcacaggttctcacagaagttgtttactccaatggcggtttctatatgaa**

**MK603150.1 BtCoV alpha RIBSP-K** **caacttgttgtagcaatactgatcgttattatagactttgcaatgagcttgcacaggttctcacagaagttgtttactccaatggcggtttctatatgaa**

**gb:GQ259976 BtCoV M.das/VM2/NL** **ctacatgctgtagcaatactgatcgttattatagactgtgtaatgagcttgcgcaggtgcttacagaagtggtttactccaatggtggattttatatgaa**

**MG310240.1 alpha BtCoV/2015092** **aaaattgttgtactagctcagatcgttattatagactatgtaacgagttagcacaagttctaactgaagtggtctactctaacggcggtttttatttgaa**

**MG310244.1 alpha BtCoV/2015081** **ctacgtgttgtacatcaagcgaaaagttttaccgacttagcaatgaacttgctcaggttttgaccgaggtagtttactccaatggaggattctacataaa**

110 120 130 140 150 160 170 180 190 200

....|....|....|....|....|....|....|....|....|....|....|....|....|....|....|....|....|....|....|....|

**gb:KU131210 BatCoV/NIG/2011/13** **accaggtggcactagtagtggtgattcaacaactgcatacgctaatagtgtgtttaatatatgtcaggccgttagtgcaaacattaatacattactatct**

**gb:KU131214 BatCoV/NGR/E.heivu** **accaggtggcactagtagtggtgattcaacaactgcatacgctaatagtgtgtttaatatatgtcaggccgttagtgcaaacattaatacattactatct**

**gb:KU131215 BatCoV/NGR/E.heivu** **accaggtggcactagtagtggtgattcaacaactgcatacgctaatagtgtgtttaatatatgtcaggccgttagtgcaaacattaatacattactatct**

**gb:KU131211 BatCoV/NGR/2011/13** **acctggtggcactagtagtggtgattcaacaactgcatacgctaatagtgtatttaatatatgtcaggccgttagtgcaaacattaatacattactatct**

**gb:KU131212 BatCoV/NGR/2011/13** **acctggtggcactagtagtggtgattcaacaactgcatacgctaatagtgtgtttaatatatgtcaggccgttagtgcaaacattaatacattactatct**

**GU065432.1 BtCoV/KEN/E.heivum/** **acctggtggcactagtagtggtgattcaacaactgcatacgctaatagtgtgtttaatatatgtcaggccgttagtgcaaacattaatacattactatct**

**GU065431.1 BtCoV/KEN/E.heivum/** **acctggtggcactagtagtggtgattcaacaactgcatacgctaatagtgtgtttaatatatgtcaggccgttagtgcaaacattaatacattactatct**

**gb:KU131213 BatCoV/NGR/E.heivu** **acctggtggcactagtagtggtgattcaacaactgcatacgctaatagtgtgtttaatatatgtcaggccgttagtgcaaacattaatacattactatct**

**GU065384.1 BtCoV/KEN/E. heivum** **acctggtggcactagtagtggtgattcaacaactgcatacgctaatagtgtgtttaatatatgtcaggccgttagcgcaaacattaatacattactatct**

**GU065442.1 BtCoV/KEN/E. heivum** **acctggtggcactagtagtggtgattcaacaactgcatacgctaatagtgtgtttaatatatgtcaggccgttagcgcaaacattaatacattactatct**

**GU065395.1 Bt CoV/KEN/E. heivu** **acctggtggcactagtagtggtgattcaacaactgcatacgctaatagtgtgtttaatatatgtcaggccgttagcgcaaacattaatacattactatct**

**GU065377.1 BtCoV/KEN/E.heivum/** **acctggtggcactagtagtggtgattcaacaactgcatacgctaatagtgtgtttaatatatgtcaggccgttagcgcaaacattaatacattactatct**

**GU065378.1 BtCoV/KEN/E.heivum/** **acctggtggcactagtagtggtgattcaacaactgcatacgctaatagtgtgtttaatatatgtcaggccgttagcgcaaacattaatacattactatct**

**GU065437.1 BtCoV/KEN/H.heivum/** **acctggtggcactagtagtggtgattcaacgactgcatacgctaatagtgtttttaatatatgtcaggccgttagcgcaaacattaatacattactatct**

**gb:KX284999 /PREDICT-GVF-CM-EC** **acctggtggtacaagtagtggtgattccaccactgcttatgcaaatagtgtgtttaatatatgccaggctgtcagtgctaacattaatactttgttatcc**

**gb:KX285001 PREDICT-GVF-CM-ECO** **acctggtggtacaagtagtggtgattccaccactgcttatgcaaatagtgtgtttaatatatgccaggctgtcagtgctaacattaatactttgttatcc**

**gb:KX285008 PREDICT-GVF-CM-ECO** **acctggtggtacaagtagtggtgattccaccactgcttatgcaaatagtgtgtttaatatatgccaggctgtcagtgctaacattaatactttgttatcc**

**gb:KX285009 PREDICT-GVF-CM-ECO** **acctggtggtacaagtagtggtgattccaccactgcttatgcaaatagtgtgtttaatatatgccaggctgtcagtgctaacattaatactttgttatcc**

**gb:KX285012 PREDICT-GVF-CM-ECO** **acctggtggtacaagtagtggtgattccaccactgcttatgcaaatagtgtgtttaatatatgccaggctgtcagtgctaacattaatactttgttatcc**

**gb:KX285023 PREDICT-GVF-CM-ECO** **acctggtggtacaagtagtggtgattccaccactgcttatgcaaatagtgtgtttaatatatgccaggctgtcagtgctaacattaatactttgttatcc**

**gb:KX284994 PREDICT-GVF-CM-ECO** **acctggtggtacaagtagtggtgattccaccactgcttatgcaaatagtgtgtttaatatatgccaggctgtcagtgctaacattaatactttgttatcc**

**gb:KX284993 PREDICT-GVF-CM-ECO** **acctggtggtacaagtagtggtgattccaccactgcttatgcaaatagtgtgtttaatatatgccaggctgtcagtgctaacattaatactttgttatcc**

**gb:KX284951 PREDICT-GVF-CM-ECO** **acctggtggtacaagtagtggtgattccaccactgcttatgcaaatagtgtgtttaatatatgccaggctgtcagtgctaacattaatactttgttatcc**

**gb:KX284954 PREDICT-GVF-CM-ECO** **acctggtggtacaagtagtggtgattccaccactgcttatgcaaatagtgtgtttaatatatgccaggctgtcagtgctaacattaatactttgttatcc**

**gb:KX285006 PREDICT-GVF-CM-ECO** **acctggtggtacaagtagtggtgattccaccactgcttatgcaaatagtgtgtttaatatatgccaggctgtcagtgctaacattaatactttgttatcc**

**gb:KX285007 :PREDICT-GVF-CM-EC** **acctggtggtacaagtagtggtgattccaccactgcttatgcaaatagtgtgtttaatatatgccaggctgtcagtgctaacattaatactttgttatcc**

**gb:KX284989 PREDICT-GVF-CM-ECO** **acctggtggtacaagtagtggtgattccaccactgcttatgcaaatagtgtgttcaatatatgccaggctgtcagtgctaacattaatactttgttatcc**

**gb:KX284990 PREDICT-GVF-CM-ECO** **acctggtggtacaagtagtggtgattccaccactgcttatgcaaatagtgtgttcaatatatgccaggctgtcagtgctaacattaatactttgttatcc**

**gb:KX284986 PREDICT-GVF-CM-ECO** **acctggtggtacaagtagtggtgattccaccactgcttatgcaaatagtgtgttcaatatatgccaggctgtcagtgctaacattaatactttgttatcc**

**gb:KX284987 PREDICT-GVF-CM-ECO** **acctggtggtacaagtagtggtgattccaccactgcttatgcaaatagtgtgttcaatatatgccaggctgtcagtgctaacattaatactttgttatcc**

**gb:KX285075 PREDICT-CD115912/D** **acctggtggtacaagtagtggtgattctactactgcttatgcaaatagtgtgtttaatatatgccaggctgtcagtgctaacattaatactttgttatcc**

**gb:KX285076 PREDICT-CD115914/d** **acctggtggtacaagtagtggtgattctactactgcttatgcaaatagtgtgtttaatatatgccaggctgtcagtgctaacattaatactttgttatcc**

**gb:KX285024 /PREDICT-GVF-CM-EC** **acctggtggtacaagtagtggtgattcaaccactgcttatgcaaatagtgtgtttaatatatgccaggctgtcagtgctaacattaatactttgttatcc**

**gb:KX285025 PREDICT-GVF-CM-ECO** **acctggtggtacaagtagtggtgattcaaccactgcttatgcaaatagtgtgtttaatatatgccaggctgtcagtgctaacattaatactttgttatcc**

**gb:KX284957 PREDICT-GVF-CM-ECO** **acctggtggtacaagtagtggtgattcaaccactgcttatgcaaatagtgtgtttaatatatgccaggctgtcagtgctaacattaatactttgttatcc**

**gb:KX284958 PREDICT-GVF-CM-ECO** **acctggtggtacaagtagtggtgattcaaccactgcttatgcaaatagtgtgtttaatatatgccaggctgtcagtgctaacattaatactttgttatcc**

**gb:KX284985 PREDICT-GVF-CM-ECO** **acctggtggtacaagtagtggtgattcaaccactgcttatgcaaatagtgtgtttaatatatgccaggctgtcagtgctaacattaatactttgttatcc**

**gb:KX285427 PREDICT-130518Bt34** **acctggtggtacaagtagtggtgactctaccactgcttatgcaaatagtgtgtttaatatatgtcaggccgtcagtgctaacattaatactttgttatcc**

**gb:KX285428 PREDICT-130518Bt3/** **acctggtggtacaagtagtggtgactctaccactgcttatgcaaatagtgtgtttaatatatgtcaggccgtcagtgctaacattaatactttgttatcc**

**gb:KX285429 PREDICT-130518Bt35** **acctggtggtacaagtagtggtgactctaccactgcttatgcaaatagtgtgtttaatatatgtcaggccgtcagtgctaacattaatactttgttatcc**

**gb:KX285431 PREDICT-140403Bt16** **acctggtggtacaagtagtggtgactctaccactgcttatgcaaatagtgtgtttaatatatgtcaggccgtcagtgctaacattaatactttgttatcc**

**gb:KX285099 PREDICT-CD116096/D** **acctggtggtacaagtagtggtgattctaccactgcttatgcaaatagtgtgtttaatatatgtcaggccgtcagtgctaacattaatactttgttatcc**

**gb:KX285077 PREDICT-CD115937/D** **acctggtggtacaagtagtggtgattctaccactgcttatgcaaacagtgtgtttaatatatgtcaggctgtcagtgctaacattaatactttgttatct**

**gb:KX285078 PREDICT-CD115938/d** **acctggtggtacaagtagtggtgattctaccactgcttatgcaaacagtgtgtttaatatatgtcaggctgtcagtgctaacattaatactttgttatct**

**gb:KX285080 PREDICT-CD115941/D** **acctggtggtacaagtagtggtgattctaccactgcttatgcaaacagtgtgtttaatatatgtcaggctgtcagtgctaacattaatactttgttatct**

**gb:KX285082 PREDICT-CD115947/D** **acctggtggtacaagtagtggtgattctaccactgcttatgcaaacagtgtgtttaatatatgtcaggctgtcagtgctaacattaatactttgttatct**

**gb:KX285085 PREDICT-CD115956/D** **acctggtggtacaagtagtggtgattctaccactgcttatgcaaacagtgtgtttaatatatgtcaggctgtcagtgctaacattaatactttgttatct**

**gb:KX285087 PREDICT-CD116004/D** **acctggtggtacaagtagtggtgattctaccactgcttatgcaaatagtgtgtttaatatatgtcaggctgtcagtgctaacattaatactttgttatct**

**gb:KX285091 PREDICT-CD116015/D** **acctggtggtacaagtagtggtgattctaccactgcttatgcaaatagtgtgtttaatatatgtcaggctgtcagtgctaacattaatactttgttatct**

**gb:KX285088 PREDICT-CD116006/D** **acctggtggtacaagtagtggtgattctaccactgcttatgcaaatagtgtgtttaatatatgtcaggctgtcagtgctaacattaatactttgttatct**

**gb:KX285070 PREDICT-CD115124A/** **acctggaggtacaagtagtggtgattctaccactgcttatgcaaatagtgtgtttaatatatgtcaggctgtcagtgctaacattaatactttgttatcc**

**gb:KX285071 PREDICT-CD115222/D** **acctggaggtacaagtagtggtgattctaccactgcttatgcaaatagtgtgtttaatatatgtcaggctgtcagtgctaacattaatactttgttatcc**

**gb:KX285086 PREDICT-CD115975/D** **acctggaggtacaagtagtggtgattctaccactgcttatgcaaatagtgtgtttaatatatgtcaggctgtcagtgctaacattaatactttgttatcc**

**gb:KX285100 PREDICT-CD116101/D** **acctggaggtacaagtagtggtgattctaccactgcttatgcaaatagtgtgtttaatatatgtcaggctgtcagtgctaacattaatactttgttatcc**

**gb:KX285105 PREDICT-CD116107/D** **acctggaggtacaagtagtggtgattctaccactgcttatgcaaatagtgtgtttaatatatgtcaggctgtcagtgctaacattaatactttgttatcc**

**gb:KX285081 PREDICT-CD115943/D** **acctggaggtacaagtagtggtgattctaccactgcttatgcaaatagtgtgtttaatatatgtcaggctgtcagtgctaacattaatactttgttatcc**

**gb:KX285101 PREDICT-CD116102/D** **acctggaggtacaagtagtggtgattctaccactgcttatgcaaatagtgtgtttaatatatgtcaggctgtcagtgctaacattaatactttgttatcc**

**gb:KX285102 PREDICT-CD116103/D** **acctggaggtacaagtagtggtgattctaccactgcttatgcaaatagtgtgtttaatatatgtcaggctgtcagtgctaacattaatactttgttatcc**

**gb:KX285103 PREDICT-CD116105/D** **acctggaggtacaagtagtggtgattctaccaatgtttatgcaaatagtgtgtttaatatatgtcaggctgtcagtgctaacattaatactttgttatcc**

**gb:MG762628 BtCoV/HKN/HKU9/Rou** **acctggtggtaccagtagtggtgactccacgactgcttatgctaacagtgtttttaacatttgtcaggctgttagtgctaaccttaataccttcttatct**

**gb:MG762630 BtCoV/HKN/HKU9/Rou** **acctggtggtaccagtagtggtgactccacgactgcttatgctaacagtgtttttaacatttgtcaggctgttagtgctaaccttaataccttcttatct**

**gb:MG762631 BtCoV/HKN/HKU9/Rou** **acctggtggtaccagtagtggtgactccacgactgcttatgctaacagtgtttttaacatttgtcaggctgttagtgctaaccttaataccttcttatct**

**gb:MG762633 BtCoV/HKN/HKU9/Rou** **acctggtggtaccagtagtggtgactccacgactgcttatgctaacagtgtttttaacatttgtcaggctgttagtgctaaccttaataccttcttatct**

**gb:MG762634 BtCoV/HKN/HKU9/Rou** **acctggtggtaccagtagtggtgactccacgactgcttatgctaacagtgtttttaacatttgtcaggctgttagtgctaaccttaataccttcttatct**

**gb:MG762635 BtCoV/HKN/HKU9/Rou** **acctggtggtaccagtagtggtgactccacgactgcttatgctaacagtgtttttaacatttgtcaggctgttagtgctaaccttaataccttcttatct**

**gb:MG762638 BtCoV/HKN/HKU9/Rou** **acctggtggtaccagtagtggtgactccacgactgcttatgctaacagtgtttttaacatttgtcaggctgttagtgctaaccttaataccttcttatct**

**gb:MG762639 BtCoV/HKN/HKU9/Rou** **acctggtggtaccagtagtggtgactccacgactgcttatgctaacagtgtttttaacatttgtcaggctgttagtgctaaccttaataccttcttatct**

**gb:MG762640 BtCoV/HKN/HKU9/Rou** **acctggtggtaccagtagtggtgactccacgactgcttatgctaacagtgtttttaacatttgtcaggctgttagtgctaaccttaataccttcttatct**

**gb:MG762641 BtCoV/HKN/HKU9/Rou** **acctggtggtaccagtagtggtgactccacgactgcttatgctaacagtgtttttaacatttgtcaggctgttagtgctaaccttaataccttcttatct**

**gb:MG762646 BtCoV/HKN/HKU9/Rou** **acctggtggtaccagtagtggtgactccacgactgcttatgctaacagtgtttttaacatttgtcaggctgttagtgctaaccttaataccttcttatct**

**gb:MG762650 BtCoV/HKN/HKU9/Rou** **acctggtggtaccagtagtggtgactccacgactgcttatgctaacagtgtttttaacatttgtcaggctgttagtgctaaccttaataccttcttatct**

**gb:MG762629 BtCoV/HKN/HKU9/Rou** **acctggtggtaccagtagtggtgattccacgactgcttatgctaacagtgtttttaacatttgtcaggctgttagtgctaaccttaataccttcttatct**

**gb:MG762643 BtCoV/HKN/HKU9/Rou** **acctggtggtaccagtagtggtgattccacgactgcttatgctaacagtgtttttaacatttgtcaggctgttagtgctaaccttaataccttcttatct**

**gb:MG762636 BtCoV/HKN/HKU9/Rou** **acctggtggtaccagtagcggtgactccacgactgcttatgctaacagtgtttttaacatttgtcaggctgttagtgctaaccttaataccttcttatct**

**gb:KX285051 BtCoV/MYS/PREDICT_** **accaggtggtacaagtagtggcgattccaccactgcatatgcaaatagtgtgtttaatatttgccaagccgttagcgctaatttaaataccttcttgtct**

**gb:KX285112 Bt/CoV/Philippines** **accaggtggtacaagcagtggcgattctactactgcatatgcaaatagtgtttttaacatatgtcaagctgttagcgctaatttgaacacctttttgtct**

**gb:KX285113 BtCoV/Philippines/** **accaggtggtacaagcagtggcgattctactactgcatatgcaaatagtgtttttaacatatgtcaagctgttagcgctaatttgaacacctttttgtct**

**gb:KX285114 BtCoV/Philippines/** **accaggtggtacaagcagtggcgattctactactgcatatgcaaatagtgtttttaacatatgtcaagctgttagcgctaatttgaacacctttttgtct**

**KP696742.1 BtCoV/MDG/ANK036F/2** **acctggtggcacctctagtggtgactcaactactgcgtatgctaatagtgtttttaatatttgtcaggccgttactgccaatttaggtactttattggct**

**KP696744.1 BtCoV/MDG/ BEM073F/** **acctggtggcacctctagtggtgactcaaccactgcgtatgctaatagtgtttttaatatttgccaggccgttactgccaatttaggtactttattggct**

**KP696747.1 BtCoV/MDG/ANK081F/2** **acctggtggcacctctagtggtgactcaaccactgcgtatgctaatagtgtttttaatatttgccaggccgttactgccaatttaggtactttattggct**

**KP696746.1 BtCoV/MDG/BEM077F/2** **acctggtggcacctctagtggtgactcaactaccgcctatgccaatagtgttttcaatatttgtcaagctgttacggctaacttaggtactttgttggct**

**KP696745.1 BtCoV/MDG/BEM074F/2** **acctggtggcacctctagtggtgactcaactaccgcctatgccaatagtgttttcaatatttgtcaagctgttacggctaacttaggtactttgttggct**

**gb:KT717381 BatCoV Art.lit/206** **acctggcggtactagtagtggcgatgctactactgcgtacgctaacagtgtttttaatatagcacaagctgtcactgcaaatgtgggggcacttatgtct**

**gb:KX285064 BtCoV PREDICT_CoV-** **ggcgggtggcactagtagcggtgatgctaccactgcttttgctaacagcgtctttaacatagcgcaggcagtcactgctaatgttggcgcattggtatct**

**gb:MG193617 BtCoV/20161014_DC1** **acctggaggtaccagtagcggagacgctactactgcatacgccaatagtgtatttaacattttgcaagcgactactgcgaatgttagtgcacttatgggt**

**gb:MG252876 BtCoV/20161014_DC1** **acctggaggtaccagtagcggagacgctactactgcatacgccaatagtgtatttaacattttgcaagcgactactgcgaatgttagtgcacttatgggt**

**gb:MG310223 BtCoV/20161014_DC_** **acctggaggtaccagtagcggagacgctactactgcatacgccaatagtgtatttaacattttgcaagcgactactgcgaatgttagtgcacttatgggt**

**gb:MG310233 BtCoV/20161011_DC1** **acctggaggtaccagtagcggagacgctactactgcatacgccaatagtgtatttaacattttgcaagcgactactgcgaatgttagtgcacttatgggt**

**gb:MG310243 BtCoV/20150816HFP_** **acctggaggtaccagtagcggagacgctactactgcatacgccaatagtgtatttaacattttgcaagcaactactgcgaatgtcagtgcacttatgggc**

**gb:MG205593 BtCoV/20150720ABA_** **acctggaggcaccagtagcggagatgctactactgcgtacgccaatagtgtttttaacattttgcaggcaactactgcaaatgtcagtgcacttatgggc**

**gb:MG310245 BtCoV/20150720ABA_** **acctggaggcaccagtagcggagatgctactactgcgtacgccaatagtgtttttaacattttgcaggcaactactgcaaatgtcagtgcacttatgggc**

**gb:MG817483 BtCoV/20140127ABA_** **acctggaggcaccagtagcggagatgctactactgcgtacgccaatagtgtttttaacattttgcaggcaactactgcaaatgtcagtgcacttatgggc**

**gb:MG310225 BtCoV/20141022HBI_** **acctggaggtaccagtagcggagatgctactactgcatacgccaatagtgtttttaacattttgcaggcaactactgcaaatgtcagtgcacttatgggc**

**gb:MG205595 BtCoV/20150816HFP_** **acctggaggtaccagtagcggagacgctactactgcatacgccaatagtgtttttaacattttgcaggcaactactgcaaatgttagtgcacttatgggc**

**gb:MG205596 BtCoV/20150816HFP_** **acctggaggtaccagtagcggagacgctactactgcatacgccaatagtgtttttaacattttgcaggcaactactgcaaatgttagtgcacttatgggc**

**gb:MG205597 BtCoV/20150816HFP_** **acctggaggtaccagtagcggagacgctactactgcatacgccaatagtgtttttaacattttgcaggcaactactgcaaatgttagtgcacttatgggc**

**gb:MG310229 BtCoV/20150819LFU_** **acctggaggtaccagtagcggagacgctactactgcatacgccaatagtgtttttaacattttgcaggcaactactgcaaatgttagtgcacttatgggc**

**gb:MG817484 BtCoV/20141103SRP** **acctggaggtaccagtagcggagatgccaccactgcatacgccaatagtgtttttaacattttgcaggcgactactgcaaatgtcagtgcacttatgggc**

**gb:MG252869 BtCoV/20160303FEK_** **acctggtggcaccagtagcggagatgctactactgcatacgccaatagtgtttttaacattttgcaggcgactactgcaaatgttagtgcacttatgggc**

**gb:MG252872 BtCoV/20160304FEK** **acctggtggcaccagtagcggagatgctactactgcatacgccaatagtgtttttaacattttgcaggcgactactgcaaatgttagtgcacttatgggc**

**gb:MG252873 BtCoV/20160304FEK_** **acctggtggcaccagtagcggagatgctactactgcatacgccaatagtgtttttaacattttgcaggcgactactgcaaatgttagtgcacttatgggc**

**gb:MG310226 BtCoV/20150105CGR_** **acctggtggcaccagtagcggagatgctactactgcatacgccaacagtgtttttaacattttgcaggcgactactgcaaatgttagtgcacttatgggc**

**gb:MG817485 BtCoV/20150105CGR_** **acctggtggcaccagtagcggagatgctactactgcatacgccaacagtgtttttaacattttgcaggcgactactgcaaatgttagtgcacttatgggc**

**gb:MG817486 BtCoV/20150105CGR_** **acctggtggcaccagtagcggagatgctactactgcatacgccaacagtgtttttaacattttgcaggcgactactgcaaatgttagtgcacttatgggc**

**gb:MG205591 BtCoV/20150106CDK_** **acctggaggtaccagtagcggagatgccacaaccgcatatgccaatagtgtttttaacattctgcaggcgactactgcaaatgttagtgcgcttatggga**

**gb:MG252862 BtCoV/20150919CDK_** **acctggaggtaccagtagcggagatgccacaaccgcatatgccaatagtgtttttaacattctgcaggcgactactgcaaatgttagtgcgcttatggga**

**gb:MG817496 BtCoV/20150919CDK2** **acctggaggtaccagtagcggagatgccacaaccgcatatgccaatagtgtttttaacattctgcaggcgactactgcaaatgttagtgcgcttatggga**

**gb:MG252859 BtCoV/20150920CGC_** **acctggaggtaccagtagcggagatgccaccaccgcatatgccaatagtgtttttaacattctgcaggcgactactgcaaatgttagtgcgcttatggga**

**gb:MG817488 BtCoV/20150107CGC_** **acctggaggtaccagtagcggagatgccaccaccgcatatgccaatagtgtttttaacattctgcaggcgactactgcaaatgttagtgcgcttatggga**

**gb:MG817494 BtCoV/20150920CGC_** **acctggaggtaccagtagcggagatgccaccaccgcatatgccaatagtgtttttaacattctgcaggcgactactgcaaatgttagtgcgcttatggga**

**gb:MG252864 BtCoV/20150108CCK_** **acctggaggtaccagtagcggagatgccaccaccgcatatgccaatagtgtttttaacattttgcaggccaccactgcaaatgttagtgcacttatgggt**

**KC776174.1 Human MERS/CoV 2c J** **acctggaggtaccagtagcggagatgccaccactgcatatgccaatagtgtctttaacattttgcaggcgacaactgctaatgtcagtgcacttatgggt**

**MG923474.1 MERS-CoV camel/Nige** **acctggaggtaccagtagcggagatgccaccactgcatatgccaatagtgtctttaacattttgcaggcgacaactgctaatgtcagtgcacttatgggt**

**gb:KF500943 BtCoV/Pipistrellus** **acctggaggaaccagtagcggagatgccacaactgcatatgccaatagcgtttttaacattttacaggcaactactgcaaatgttagcgctttgatgggc**

**gb:KF500944 BtCoV/Pipistrellus** **acctggaggaaccagtagcggagatgccacaactgcatatgccaatagcgtttttaacattttacaggcaactactgcaaatgttagcgctttgatgggc**

**gb:KF500942 BtCoV/Pipistrellus** **acctggaggaaccagtagcggagatgccacaactgcatatgccaatagcgtttttaacattttacaggcaactactgcaaatgttagcgctttgatgggc**

**gb:KF500946 BtCoV/Pipistrellus** **acctggaggaaccagtagcggagatgccaccactgcatatgccaatagcgtctttaacattttacaggcaactactgcaaatgttagcgctttgatgggc**

**gb:KF500947 BtCoV/Pipistrellus** **acctggaggaaccagtagcggagatgccaccactgcatatgccaatagcgtctttaacattttacaggcaactactgcaaatgttagcgctttgatgggc**

**gb:HQ184059 BatCoV/H.sav/J/Spa** **acccggtggaaccagtagcggagatgccacaactgcatatgccaatagcgttttcaacattttgcaggcgactactgcaaatgtaagcgctttgatgggc**

**gb:KT717386 BatCoV Eum.gla/242** **acctggtggtaccagtagcggagatgccactactgcatatgccaatagtgtttttaacatcttgcaagcgactactgcaaatgttagcgctttaattggc**

**gb:GQ259977 BtCoV P.pipi/VM314** **acctggtggtacgagtagtggagatgccacaactgcatatgccaacagtgtgttcaacattttgcaagcgactacagcaaatgttagcgcactcatgggt**

**gb:KX285197 BtCoV/CHN/HKU5/PRE** **acctggtgggaccagtagcggcgatgcaaccactgcttacgccaatagtgtctttaacattttgcaggcgactaccgcaaatgttagtgcacttatgggc**

**gb:KX285199 BtCoV/CHN/HKU5/PRE** **acctggcgggaccagtagcggcgatgcaaccactgcttacgccaatagtgtctttaacattctgcaggcgacgactgcgaatgttagtgcacttatgggc**

**gb:KX285200 BtCoV/CHN/HKU5/PRE** **acctggtgggactagtagcggcgatgcaaccactgcttacgccaatagtgtttttaacattctgcaggcgacaaccgcgaatgttagtgcacttatgggc**

**gb:HQ184062 BtCoV/E.isa/M/Spai** **gcctggtggcacaagcagtggtgacgccactacagcatatgccaatagtgttttcaacattctgcaagcgactactgcgaatgtgagtgcacttatggga**

**gb:KC522038 BtCoV/HKN/Tylonyct** **accaggtggtacgagtagtggtgatgcaactactgcttatgccaatagtgtttttaacatattacaagccaccaccgctaatgttagtgcacttatgagt**

**gb:KC522039 BtCoV/HKN/Tylonyct** **accaggtggtacgagtagtggtgatgcaactactgcttatgccaatagtgtttttaacatattacaagccaccaccgctaatgttagtgcacttatgagt**

**gb:KC522047 BtCoV/HKN/Tylonyct** **accaggtggtacgagtagtggtgatgcaactactgcttatgccaatagtgtttttaacatattacaagccaccaccgctaatgttagtgcacttatgagt**

**gb:KC522042 BtCoV/HKN/Tylonyct** **accaggtggtacgagtagtggtgatgcaactactgcttatgccaatagtgtttttaacatattacaagccaccaccgctaatgttagtgcacttatgagt**

**gb:KC522043 BtCoV/HKN/Tylonyct** **accaggtggtacgagtagtggtgatgcaactactgcttatgccaatagtgtttttaacatattacaagccaccaccgctaatgttagtgcacttatgagt**

**gb:KC522036 BtCoV/HKN/Tylonyct** **accaggtggtacgagtagtggtgatgcaactactgcttatgccaatagtgtttttaacatattacaagccaccaccgctaatgttagtgcacttatgagt**

**gb:KC522037 BtCoV/HKN/Tylonyct** **accaggtggtacgagtagtggtgatgcaactactgcttatgccaatagtgtttttaacatattacaagccaccaccgctaatgttagtgcacttatgagt**

**gb:KX447563 BatSARS/CHN/HKU3/1** **accaggtggaacatcatccggtgatgccacaactgcttatgctaatagtgtgtttaacatctgtcaagcagtaacagctaatgtaaatgcacttctctca**

**gb:KX447564 BatSARS/CHNHKU3/15** **accaggtggaacatcatccggtgatgccacaactgcttatgctaatagtgtgtttaacatctgtcaagcagtaacagctaatgtaaatgcacttctctca**

**gb:MG772855 BtCoV/CHN/SARS-lik** **accaggtggaacatcatccggtgatgccacaactgcttatgctaatagtgtgtttaacatctgtcaagcagtaacagctaatgtaaatgcacttctttca**

**gb:MG772880 BtCoV/CHN/SARS-lik** **accaggtggaacatcatccggtgatgccacaactgcttatgctaatagtgtgtttaacatctgtcaagcagtaacagctaatgtaaatgcacttctttca**

**gb:MG772855 SARS-like bat-SL-C** **accaggtggaacatcatccggtgatgccacaactgcttatgctaatagtgtgtttaacatctgtcaagcagtaacagctaatgtaaatgcacttctttca**

**gb:MG772883 BtCoV/CHN/SARS-lik** **accaggtggaacatcatccggtgatgccacaactgcttatgctaatagtgtgtttaacatctgccaagcagtaacagctaatgtaaatgcacttctttca**

**gb:MG772862 BtCoV/CHNSARS-like** **accaggtggaacatcacccggtgatgccacaactgcttatgctaatagtgtgtttaacatctgccaagcagtaacagctaatgtaaatgcacttctttca**

**gb:MG772879 SARS-like bat-SL-C** **accaggtggaacatcatccggtgatgccacaactgcttatgctaataatgtgtttaacatctgccaagcagtaacagctaatgtaaatgcacttctttca**

**gb:MG772858 BtCoV/CHN/SARS-lik** **accaggtggaacatcatccggtgatgccacaactgcttatgctaatagtgtgtttaacatctgtcaagcagtaacagctaaagtaaatgcacttctttca**

**gb:MG772859 SARS-like bat-SL-C** **accaggtggaacatcatccggtgatgccacaactgcttatgctaatagtgtgtttaacatctgtcaagcagtaacagctaaagtaaatgcacttctttca**

**gb:MG772858 SARS-like bat-SL-C** **accaggtggaacatcatccggtgatgccacaactgcttatgctaatagtgtgtttaacatctgtcaagcagtaacagctaaagtaaatgcacttctttca**

**gb:KX285125 BatSARS/CHN/HKU3/P** **accaggtggaacgtcatccggtgatgccacaactgcttatgctaatagtgtgtttaacatctgtcaagcagtaacagctaatgtgaatgcacttctctca**

**gb:KX285125 BtCoV/SARS/CHN/HKU** **accaggtggaacgtcatccggtgatgccacaactgcttatgctaatagtgtgtttaacatctgtcaagcagtaacagctaatgtgaatgcacttctctca**

**gb:MG772861 SARS-like bat-SL-C** **accaggtggaacgtcatccggtgatgccacaactgcttatgctaatagtgtgtttaacatctgtcaagcagtaacagctaatgtgaatgcacttctctca**

**gb:MG772852 BtCoV/CHN/SARS-lik** **accaggtggaacgtcatccggtgatgccacaactgcttatgctaatagtgtgtttaacatctgtcaagcagtaacagctaatgtgaatgcacttctctca**

**gb:MG772875 BtCoV/CHNSARS-like** **accaggtggaacgtcatccggtgatgccacaactgcttatgctaatagtgtgtttaacatctgtcaagcagtaacagctaatgtgaatgcacttctctca**

**gb:MG772874 BtCoV/CHN/SARS-lik** **accaggtggaacgtcatccggtgatgccacaactgcttatgctaatagtgtgtttaacatctgtcaagcagtaacagctaatgtgaatgcacttctctca**

**gb:MG772865 BtCoV/CHN/SARS-lik** **accaggtggaacgtcatccggtgatgccacaactgcttatgctaatagtgtgtttaacatctgtcaagcagtaacagctaatgtgaatgcacttctctca**

**gb:MG772863 BtCoV/CHN/SARS-lik** **accaggtggaacgtcatccggtgatgccacaactgcttatgctaatagtgtgtttaacatctgtcaagcagtaacagctaatgtgaatgcacttctctca**

**gb:MG772891 BtCoV/CHN/SARS-lik** **accaggtggaacgtcatccggtgatgccacaactgcttatgctaatagtgtgtttaacatctgtcaagcagtaacagctaatgtgaatgcacttctctca**

**gb:MG772891 SARS-like bat-SL-C** **accaggtggaacgtcatccggtgatgccacaactgcttatgctaatagtgtgtttaacatctgtcaagcagtaacagctaatgtgaatgcacttctctca**

**gb:MG772890 BtCoV/CHN/SARS-lik** **cccaggtggaacgtcatccggtgatgccacaactgcttatgctaatagtgtgtttaacatctgtcaagcagtaacagctaatgtgaatgcacttctctca**

**gb:MG772890 Bat SARS-like bat-** **cccaggtggaacgtcatccggtgatgccacaactgcttatgctaatagtgtgtttaacatctgtcaagcagtaacagctaatgtgaatgcacttctctca**

**gb:MG772854 BtCoV/CHN/SARS-lik** **accaggtggaacgtcatccggtgatgccacaactgcttatgctaatagtgtgtttaacatctgtcaagcagtaaaagctaatgtgaatgcacttctctca**

**gb:MG772854 SARS-like bat-SL-C** **accaggtggaacgtcatccggtgatgccacaactgcttatgctaatagtgtgtttaacatctgtcaagcagtaaaagctaatgtgaatgcacttctctca**

**gb:MG772849 SARS-like bat-SL-C** **accaggtggaacgtcatccggtgatgccacaactgcttatgctaatagtgtgttaaacatctgtcaagcagtaacagctaatgtgaatgcacttctctca**

**gb:MG772848 SARS-like bat-SL-C** **accaggtggaacgtcatccggggatgccacaactgcttatgctaatagtgtgtttaacatctgtcaagcagtaacagctaatgtgaatgcacttctctca**

**gb:MG772884 BtCoV/CHN/SARS-lik** **accaggtggaacgtcatccggtgatgccacaaatgcttatgctaatagtgtgtttaacatctgtcaagcagtaacagctaatgtgaattcacttctctca**

**gb:KY423420 BtCoV/FRA_EPI1_387** **accaggaggtacatcttcaggagatgctaccactgcttatgctaatagcgtttttaacatttgccaagctgttacagccaatgttaatgcgcttttgtct**

**gb:KY423421 BtCoVFRA_EPI1_3871** **accaggaggtacatcttcaggagatgctaccactgcttatgctaatagcgtttttaacatttgccaagctgttacagccaatgttaatgcgcttttgtct**

**gb:KY423431 BtCoV/FRA_EPI1_387** **accaggaggtacatcttcaggagatgctaccactgcttatgctaatagcgtttttaacatttgccaagctgttacagccaatgttaatgcgcttttgtct**

**gb:KY423422 BtCoV/FRA_EPI1_387** **accaggaggtacatcttcaggagatgctaccactgcttatgctaatagcgtttttaacatttgccaagctgttacagccaatgttaatgcgcttttgtct**

**gb:KY423428 BtCoV/FRA_EPI1_388** **accaggaggtacatcttcaggagatgctaccactgcttatgctaatagcgtttttaacatttgccaagctgttacagccaatgttaatgcgcttttgtct**

**gb:KY423425 BtCoV/FRA_EPI1_388** **accaggaggtacatcttcaggagatgctaccactgcttatgctaatagcgtttttaacatttgccaagctgttacagccaatgttaatgcgcttttgtct**

**gb:KY423430 BtCoV/FRA_EPI1_389** **accaggaggtacatcttcaggagatgctaccactgcttatgctaatagcgtttttaacatttgccaagctgttacagccaatgttaatgcgcttttgtct**

**gb:KY423424 BtCoV/FRA_EPI1_389** **accaggaggtacatcttcaggagatgctaccactgcttatgctaatagcgtttttaacatttgccaagctgttacagccaatgttaatgcgcttttgtct**

**gb:KY423432 BtCoV/FRA_EPI1_390** **accaggaggtacatcttcaggagatgctaccactgcttatgctaatagcgtttttaacatttgccaagctgttacagccaatgttaatgcgcttttgtct**

**gb:KY423419 BtCoV/FRA_EPI1_394** **accaggaggtacatcttcaggagatgctaccactgcttatgctaatagcgtttttaacatttgccaagctgttacagccaatgttaatgcgcttttgtct**

**gb:KY423427 BtCoV/FRA_EPI1_400** **accaggaggtacatcttcaggagatgctaccactgcttatgctaatagcgtttttaacatttgccaagctgttacagccaatgttaatgcgcttttgtct**

**gb:KY423417 BtCoV/FRA_EPI1_401** **accaggaggtacatcttcaggagatgctaccactgcttatgctaatagcgtttttaacatttgccaagctgttacagccaatgttaatgcgcttttgtct**

**gb:KY423397 FRA_EPI1_3875_3E_P** **accaggaggtacatcttcaggagatgctaccactgcttatgctaatagcgtttttaacatttgccaagctgttacagccaatgttaatgcgcttttgtct**

**gb:KY423398 BtCoV/FRA_EPI1_388** **accaggaggtacatcttcaggagatgctaccactgcttatgctaatagcgtttttaacatttgccaagctgttacagccaatgttaatgcgcttttgtct**

**gb:KY423399 BtCoV/FRA_EPI1_393** **accaggaggtacatcttcaggagatgctaccactgcttatgctaatagcgtttttaacatttgccaagctgttacagccaatgttaatgcgcttttgtct**

**gb:KY423411 BtCoV/FRA_EPI1_394** **accaggaggtacatcttcaggagatgctaccactgcttatgctaatagcgtttttaacatttgccaagctgttacagccaatgttaatgcgcttttgtct**

**gb:KY423400 BtCoV/FRA_EPI1_396** **accaggaggtacatcttcaggagatgctaccactgcttatgctaatagcgtttttaacatttgccaagctgttacagccaatgttaatgcgcttttgtct**

**gb:KY423396 BtCoV/FRA_EPI1_400** **accaggaggtacatcttcaggagatgctaccactgcttatgctaatagcgtttttaacatttgccaagctgttacagccaatgttaatgcgcttttgtct**

**gb:KY423395 BtCoV/FRA_EPI1_401** **accaggaggtacatcttcaggagatgctaccactgcttatgctaatagcgtttttaacatttgccaagctgttacagccaatgttaatgcgcttttgtct**

**gb:KY502395 SARS-rel/BtCoV/Rhi** **accaggaggtacatcttcaggagatgctaccactgcttatgctaatagcgtttttaacatttgccaagctgttacagccaatgttaatgcgcttttgtct**

**gb:KY423402 BtCoV/FRA_EPI1_387** **accaggaggtacatcttcaggagatgctaccactgcttatgctaatagcgtttttaacatttgccaagctgttacagccaatgttaatgcgcttttgtct**

**gb:KY423403 BtCoV/FRA_EPI1_Rhf** **accaggaggtacatcttcaggagatgctaccactgcttatgctaatagcgtttttaacatttgccaagctgttacagccaatgttaatgcgcttttgtct**

**gb:KY423388 BtCoV/FRA_EPI1_Rhf** **accaggaggtacatcttcaggagatgctaccactgcttatgctaatagcgtttttaacatttgccaagctgttacagccaatgttaatgcgcttttgtct**

**gb:KY423389 BtCoV/FRA_EPI1_Rhf** **accaggaggtacatcttcaggagatgctaccactgcttatgctaatagcgtttttaacatttgccaagctgttacagccaatgttaatgcgcttttgtct**

**gb:KY423390 BtCoV/FRA_EPI1_Rhf** **accaggaggtacatcttcaggagatgctaccactgcttatgctaatagcgtttttaacatttgccaagctgttacagccaatgttaatgcgcttttgtct**

**gb:KY423394 BtCoV/FRA_EPI1_Rhf** **accaggaggtacatcttcaggagatgctaccactgcttatgctaatagcgtttttaacatttgccaagctgttacagccaatgttaatgcgcttttgtct**

**gb:KY423392 BtCoV/FRA_EPI1_Rhf** **accaggaggtacatcttcaggagatgctaccactgcttatgctaatagcgtttttaacatttgccaagctgttacagccaatgttaatgcgcttttgtct**

**gb:KY423391 BtCoV/FRA_EPI1_Rhf** **accaggaggtacatcttcaggagatgctaccactgcttatgctaatagcgtttttaacatttgccaagctgttacagccaatgttaatgcgcttttgtct**

**gb:KY423386 BtCoV/FRA_EPI1_Rhf** **accaggaggtacatcttcaggagatgctaccactgcttatgctaatagcgtttttaacatttgccaagctgttacagccaatgttaatgcgcttttgtct**

**gb:KY423412 BtCoV/SPA_EPI1_Rhf** **accaggaggtacatcttcaggagatgctaccactgcttatgctaatagcgtttttaacatttgccaagctgttacagccaatgtcaatgcgcttttgtct**

**gb:KY423375 BtCoV/FRA_EPI1_Rhf** **accaggaggtacatcttcaggagatgctaccactgcttatgcgaatagcgtttttaacatttgccaagctgttacagccaatgttaatgcgcttttgtct**

**gb:KY423413 BtCoV/SPA_EPI1_Rhf** **accaggaggtacatcttcaggagatgctaccactgcttatgccaatagcgtgtttaacatttgccaagctgttacagccaatgtcaatgcgcttttgtct**

**gb:KY423433 BtCoV/FRA_EPI1_396** **accaggaggtacatcttcaggagatgctaccactgcttatgctaattgtgtgtttaacatttgccaagctgttacagccaatgttaatgcgcttttgtct**

**gb:KY423437 BtCoV/FRA_EPI1_397** **accaggaggtacatctgcgaattgtgtgtttaacatttgtgcgaattgtgtgtttaacatttgccaagctgttacagccaatgttaatgcgcttttgtct**

**gb:KY423438 BtCoV/FRA_EPI1_396** **accaggtgcgaattgtgtgtttaacatttgccaagctgttgcgaattgtgtgtttaacatttgccaagctgttacagccaatgttaatgcgcttttgtct**

**gb:KC633214 BtCoV/Rhi_hip/R13-** **gcccggtggtacgtcatcaggtgatgctaccaccgcgtatgctaatagtgtgtttaacatttgtcaggctgttactgctaatgtaaacgcaattctttca**

**gb:KC633213 BtCoV/Rhi_hip/R46-** **acccggtggtacgtcatcaggtgatgctaccaccgcgtatgctaatagtgtgtttaacatttgtcaggctgttactgctaatgtaaacgcaattctttca**

**gb:KC633212 BtCoV/Rhi_hip/R7-0** **acccggtggtacgtcatcaggtgatgctaccaccgcgtatgctaatagtgtgtttaacatttgtcaggctgttactgctaatgtaaacgcaattctttca**

**gb:KC633209 BtCoV/Rhi_hip/R8-0** **acccggtggtacgtcatcaggtgatgctaccaccgcgtatgctaatagtgtgtttaacatttgtcaggctgttactgctaatgtaaacgcaattctttca**

**gb:KC633210 BtCoV/Rhi_hip/R77-** **acccggtggtacgtcatcaggtgatgctaccaccgcgtatgctaatagtgtgtttaacatttgtcaggctgttactgctaatgtaaacgcaattctttca**

**FJ710047.1 BtCoV/Ghana Kwam/20** **accaggtggaacgtcctctggtgatgcaaccacagcatatgctaactctgttttcaatatttgtcaggctgttagcgctaatattagtgcaatgctgtca**

**FJ710054.1 BtCoV Hipposideros/** **accaggtggaacgtcctctggtgatgcaaccacagcatatgctaactctgttttcaatatttgtcaggctgttagcgctaatattagtgcaatgctgtca**

**gb:HQ166910 ZBCoV**  **accaggtggtacatcatcaggtgacgcaactaccgcatatgctaattctgttttcaatatttgtcaggctgttagcgctaacattagtgctatgctttct**

**gb:EU834950 BtCoV R.aur/Austra** **accgggcggaacctcctccggtgacgcaaccactgcatatgctaatagcgttttcaatttatgtcaggcagttacatctaatataggtgctctgatggca**

**KP112152.1 HuCoV HKU1/HCOV/KEN** **gcctggtggtactagcagtggtgatgcaactactgcttttgctaattctgtttttaatatatgtcaggctgttactgctaacgtttgttctcttatggcc**

**KP112168.1 HuCoV/OC43 HCOV/KEN** **gcctggtggcactagtagtggtgatgcaactactgcttttgctaattcagtctttaacatatgtcaagctgtttcagccaatgtatgtgccttaatgtcg**

**KP112167.1 UNVERIFIED: HuCoV/O** **gcctggtggcactagtagtggtgatgcaactactgcttttgctaattcagtctttaacatatgtcaagctgtttcagccaatgtatgtgccttaatgtcg**

**KP112165.1 HuCoV/OC43 HCOV/KEN** **gcctggtggcactagtagtggtgatgcaactactgcttttgctaattcagtctttaacatatgtcaagctgtttcagccaatgtatgtgccttaatgtcg**

**KP112163.1 HuCoV OC43 HCOV/KEN** **gcctggtggcactagtagtggtgatgcaactactgcttttgctaattcagtctttaacatatgtcaagctgtttcagccaatgtatgtgccttaatgtca**

**KP112161.1 HuCoV OC43 HCOV/KEN** **accgggcggcaccagcagcggcgatgcgaccaccgcgtttgcgaacagcgtgtttaacatttgccaggcggtgagcgcgaacgtgtgcgcgctgatgagc**

**KC886322.1 BatCoV P.davyi49/Me** **acctggtggtactagcagtggagacsctacaacagcttatgctaatagcgttttcaacatttgtcaagctgtaactgctaatgttagtgctcttatggcc**

**MG310257.1 alpha BtCoV/2014011** **acctggtggtactacttctggtgatgctactactgcttatgcaaattctgtttttaatatcttccaagctgttagtgctaacattaacaagttgcttact**

**MG817498.1 alpha BtCoV/2015010** **acctggtggtactacttctggtgatgctactactgcttatgcaaattctgtttttaacatcttccaagctgttagtgctaacattaacaagttgcttact**

**MG310246.1 alpha BtCoV/2015010** **acctggtggtactacctctggtgatgctactactgcttatgcaaattctgtttttaacatcttccaagctgttagtgctaacattaacaagttgcttact**

**MG844332.1 alpha BtCoV/2014092** **acctggtggtactacttctggtgatgctactactgcttatgcaaattctgtttttaacatcttccaggctgttagtgctaacattaacaagttgcttact**

**gb:GQ259961 BtCoV N.noc/VM176/** **gcctggtggtacgacatctggtgatgcaacaactgcttatgcaaattctgtttttaacatttttcaagctgttagtgctaacattaacaggttgcttggt**

**gb:GQ259968 BtCoV M.das/VM105/** **gccaggtggtaccacatcaggtgatgctactactgcctatgccaattctgtctttaacatatttcaggctgtaagtgccaatgttaataggttgttaggt**

**gb:GQ259969 BtCoV M.das/VM62/N** **gccaggtggtaccacatcaggtgatgctactactgcctatgccaattctgtctttaacatatttcaggctgtaagtgccaatgttaataggttgttaggt**

**gb:GQ259970 BtCoV M.das/VM73/N** **gccaggtggtaccacatcaggtgatgctactactgcctatgccaattctgtctttaacatatttcaggctgtaagtgccaatgttaataggttgttaggt**

**gb:GQ259965 BtCoV M.das/VM3/NL** **gccaggtggtaccacatcaggtgatgctactactgcctatgccaattctgtctttaacatattccaggctgtaagtgccaatgttaataggttgttgggt**

**gb:GQ259966 BtCoV M.das/VM34/N** **gccaggtggtaccacatcaggtgatgctactactgcctatgccaattctgtctttaacatattccaggctgtaagtgccaatgttaataggttgttaggt**

**gb:GQ259971 BtCoV M.dau/VM222/** **gccaggtggtaccacatcgggtgatgctactacagcttatgccaattctgtctttaacattttccaggctgtaagtgctaatgtcaatagattgctcagt**

**gb:GQ259973 BtCoV M.dau/VM361/** **gccaagtggtaccacatcgggtgacgctactacagcttatgccaattctgtctttaacattttccaggctgtaagtgctaatgtcaatagattgctcagt**

**MK603153.1 alpha BtCoV RIBSP-K** **acctggtggtaccacttcaggtgatgcgaccacagcatacgcaaattctgtttttaacatctttcaggctactagtgctaatattaacagacttctcagt**

**MK603159.1 alpha BtCoV RIBSP-K** **acctggtggtaccacttcaggtgatgcgaccacagcatacgcaaattctgtttttaacatctttcaggctactagtgctaatattaacagacttctcagt**

**MK603152.1 alpha CoV RIBSP-KZ-** **acctggtggtaccacttcaggtgatgcgaccacagcatacgcaaattctgtttttaacatctttcaggctactagtgctaatattaacagacttctcagt**

**MK603160.1 alpha RIBSP-KZ-BatC** **acctggtggtaccacttcaggtgatgcgaccacagcatacgcaaattctgtttttaacatctttcaggctactagtgctaatattaacagacttctcagt**

**MK603156.1 BtCoV alpha RIBSP-K** **acctggtggtaccacttcaggtgatgcgaccacagcatacgcaaattctgtttttaacatctttcaggctactagtgctaatattaacagacttctcagt**

**MK603157.1 BtCoV alpha RIBSP-K** **acctggtggtactacttcaggtgatgcaaccacagcatatgcgaattctgtttttaacatttttcaggctactagtgctaatattaacagacttctcagt**

**MK603150.1 BtCoV alpha RIBSP-K** **acctggtggtactacttcaggtgatgcaaccacagcatacgcgaattctgtttttaacatttttcaggctactagtgctaatattaacagacttctcagt**

**gb:GQ259976 BtCoV M.das/VM2/NL** **gcctggaggtactacttctggtgatgccaccactgcttatgccaactcagtcttcaacatctttcaagctactagcgctaatattaacagactccttagt**

**MG310240.1 alpha BtCoV/2015092** **accaggtggtacaacatcaggtgatgcaactacagcctatgctaattcagtttttaacattttccaggcagtaagtgctaacataaatcgtattttgggc**

**MG310244.1 alpha BtCoV/2015081** **gcctggcggtactacttctggtgatgctacaactgcatatgctaattctgcttttaatattttccaagctgttagtgctaatgtaaacagattgttaact**

210 220

....|....|....|....|...

**gb:KU131210 BatCoV/NIG/2011/13** **attgatggtaataaggtgcataa**

**gb:KU131214 BatCoV/NGR/E.heivu** **attgatggtaataaggtgcataa**

**gb:KU131215 BatCoV/NGR/E.heivu** **attgatggtaataaggtgcataa**

**gb:KU131211 BatCoV/NGR/2011/13** **atagatggtaataaggtgcataa**

**gb:KU131212 BatCoV/NGR/2011/13** **atagatggtaataaggtgcataa**

**GU065432.1 BtCoV/KEN/E.heivum/** **atagatggtaataaggtgcataa**

**GU065431.1 BtCoV/KEN/E.heivum/** **atagatggtaataaggtgcataa**

**gb:KU131213 BatCoV/NGR/E.heivu** **atagatggtaataaggtgcataa**

**GU065384.1 BtCoV/KEN/E. heivum** **attgatggtaataaggtgcataa**

**GU065442.1 BtCoV/KEN/E. heivum** **attgatggtaataaggtgcataa**

**GU065395.1 Bt CoV/KEN/E. heivu** **attgatggtaataaggtgcataa**

**GU065377.1 BtCoV/KEN/E.heivum/** **attgatggtaataaggtgcataa**

**GU065378.1 BtCoV/KEN/E.heivum/** **attgatggtaataaggtgcataa**

**GU065437.1 BtCoV/KEN/H.heivum/** **attgatggtaataaggtgcataa**

**gb:KX284999 /PREDICT-GVF-CM-EC** **attgacggtaataaggtgcacaa**

**gb:KX285001 PREDICT-GVF-CM-ECO** **attgacggtaataaggtgcacaa**

**gb:KX285008 PREDICT-GVF-CM-ECO** **attgacggtaataaggtgcacaa**

**gb:KX285009 PREDICT-GVF-CM-ECO** **attgacggtaataaggtgcacaa**

**gb:KX285012 PREDICT-GVF-CM-ECO** **attgacggtaataaggtgcacaa**

**gb:KX285023 PREDICT-GVF-CM-ECO** **attgacggtaataaggtgcacaa**

**gb:KX284994 PREDICT-GVF-CM-ECO** **attgacggtaataaggtgcacaa**

**gb:KX284993 PREDICT-GVF-CM-ECO** **attgacggtaataaggtgcacaa**

**gb:KX284951 PREDICT-GVF-CM-ECO** **attgacggtaataaggtgcacaa**

**gb:KX284954 PREDICT-GVF-CM-ECO** **attgacggtaataaggtgcacaa**

**gb:KX285006 PREDICT-GVF-CM-ECO** **attgacggtaatagggtgcacaa**

**gb:KX285007 :PREDICT-GVF-CM-EC** **attgacggtaatagggtgcacaa**

**gb:KX284989 PREDICT-GVF-CM-ECO** **attgacggtaataaggtgcacaa**

**gb:KX284990 PREDICT-GVF-CM-ECO** **attgacggtaataaggtgcacaa**

**gb:KX284986 PREDICT-GVF-CM-ECO** **attgacggtaataaggtgcacaa**

**gb:KX284987 PREDICT-GVF-CM-ECO** **attgacggtaataaggtgcacaa**

**gb:KX285075 PREDICT-CD115912/D** **attgacggtaataaggtgcataa**

**gb:KX285076 PREDICT-CD115914/d** **attgacggtaataaggtgcataa**

**gb:KX285024 /PREDICT-GVF-CM-EC** **attgacggtaataaggtgcataa**

**gb:KX285025 PREDICT-GVF-CM-ECO** **attgacggtaataaggtgcataa**

**gb:KX284957 PREDICT-GVF-CM-ECO** **attgacggtaataaggtgcataa**

**gb:KX284958 PREDICT-GVF-CM-ECO** **attgacggtaataaggtgcataa**

**gb:KX284985 PREDICT-GVF-CM-ECO** **attgacggtaataaggtgcataa**

**gb:KX285427 PREDICT-130518Bt34** **attgacggcaataaggtgcataa**

**gb:KX285428 PREDICT-130518Bt3/** **attgacggcaataaggtgcataa**

**gb:KX285429 PREDICT-130518Bt35** **attgacggcaataaggtgcataa**

**gb:KX285431 PREDICT-140403Bt16** **attgacggcaataaggtgcataa**

**gb:KX285099 PREDICT-CD116096/D** **attgacggcaataaggtgcataa**

**gb:KX285077 PREDICT-CD115937/D** **attgacggcaataaggtgcataa**

**gb:KX285078 PREDICT-CD115938/d** **attgacggcaataaggtgcataa**

**gb:KX285080 PREDICT-CD115941/D** **attgacggcaataaggtgcataa**

**gb:KX285082 PREDICT-CD115947/D** **attgacggcaataaggtgcataa**

**gb:KX285085 PREDICT-CD115956/D** **attgacggcaataaggtgcataa**

**gb:KX285087 PREDICT-CD116004/D** **attgacggcaataaggtgcataa**

**gb:KX285091 PREDICT-CD116015/D** **attgacggcaataaggtgcataa**

**gb:KX285088 PREDICT-CD116006/D** **attgacggcaataaggtgcataa**

**gb:KX285070 PREDICT-CD115124A/** **attgacggcaataaggtgcataa**

**gb:KX285071 PREDICT-CD115222/D** **attgacggcaataaggtgcataa**

**gb:KX285086 PREDICT-CD115975/D** **attgacggcaataaggtgcataa**

**gb:KX285100 PREDICT-CD116101/D** **attgacggcaataaggtgcataa**

**gb:KX285105 PREDICT-CD116107/D** **attgacggcaataaggtgcataa**

**gb:KX285081 PREDICT-CD115943/D** **attgacggcaataaggtgcataa**

**gb:KX285101 PREDICT-CD116102/D** **attgacggcaataaggtgcataa**

**gb:KX285102 PREDICT-CD116103/D** **attgacggcaataaggtgcataa**

**gb:KX285103 PREDICT-CD116105/D** **attgagggcaataaggtgcataa**

**gb:MG762628 BtCoV/HKN/HKU9/Rou** **atagatggtaataagatttacac**

**gb:MG762630 BtCoV/HKN/HKU9/Rou** **atagatggtaataagatttacac**

**gb:MG762631 BtCoV/HKN/HKU9/Rou** **atagatggtaataagatttacac**

**gb:MG762633 BtCoV/HKN/HKU9/Rou** **atagatggtaataagatttacac**

**gb:MG762634 BtCoV/HKN/HKU9/Rou** **atagatggtaataagatttacac**

**gb:MG762635 BtCoV/HKN/HKU9/Rou** **atagatggtaataagatttacac**

**gb:MG762638 BtCoV/HKN/HKU9/Rou** **atagatggtaataagatttacac**

**gb:MG762639 BtCoV/HKN/HKU9/Rou** **atagatggtaataagatttacac**

**gb:MG762640 BtCoV/HKN/HKU9/Rou** **atagatggtaataagatttacac**

**gb:MG762641 BtCoV/HKN/HKU9/Rou** **atagatggtaataagatttacac**

**gb:MG762646 BtCoV/HKN/HKU9/Rou** **atagatggtaataagatttacac**

**gb:MG762650 BtCoV/HKN/HKU9/Rou** **atagatggtaataagatttacac**

**gb:MG762629 BtCoV/HKN/HKU9/Rou** **atagatggtaataagatttacac**

**gb:MG762643 BtCoV/HKN/HKU9/Rou** **atagatggtaataagatttacac**

**gb:MG762636 BtCoV/HKN/HKU9/Rou** **atagatggtaataagatttacac**

**gb:KX285051 BtCoV/MYS/PREDICT_** **atagatggtaataaaatatacac**

**gb:KX285112 Bt/CoV/Philippines** **atagatggtaacaagatttatac**

**gb:KX285113 BtCoV/Philippines/** **atagatggtaacaagatttatac**

**gb:KX285114 BtCoV/Philippines/** **atagatggtaacaagatttatac**

**KP696742.1 BtCoV/MDG/ANK036F/2** **gttgatggtaataagatctacaa**

**KP696744.1 BtCoV/MDG/ BEM073F/** **gttgatggtaataagatctacaa**

**KP696747.1 BtCoV/MDG/ANK081F/2** **gttgatggtaataagatctacaa**

**KP696746.1 BtCoV/MDG/BEM077F/2** **gttgatggtaataaatt-tacaa**

**KP696745.1 BtCoV/MDG/BEM074F/2** **gttgatggtaataaaat-tacaa**

**gb:KT717381 BatCoV Art.lit/206** **gctaatggtcataaaattgttaa**

**gb:KX285064 BtCoV PREDICT_CoV-** **accaacggccataaaataactaa**

**gb:MG193617 BtCoV/20161014_DC1** **gctaatggcaacaagattgttga**

**gb:MG252876 BtCoV/20161014_DC1** **gctaatggcaacaagattgttga**

**gb:MG310223 BtCoV/20161014_DC_** **gctaatggcaacaagattgttga**

**gb:MG310233 BtCoV/20161011_DC1** **gctaatggcaacaagattgttga**

**gb:MG310243 BtCoV/20150816HFP_** **gctaatggcaacaaaattgttga**

**gb:MG205593 BtCoV/20150720ABA_** **gctaatggcaacaaaattgttga**

**gb:MG310245 BtCoV/20150720ABA_** **gctaatggcaacaaaattgttga**

**gb:MG817483 BtCoV/20140127ABA_** **gctaatggcaacaaaattgttga**

**gb:MG310225 BtCoV/20141022HBI_** **gctaatggcaacaaaattgttga**

**gb:MG205595 BtCoV/20150816HFP_** **gctaatggcaacaaaattgttga**

**gb:MG205596 BtCoV/20150816HFP_** **gctaatggcaacaaaattgttga**

**gb:MG205597 BtCoV/20150816HFP_** **gctaatggcaacaaaattgttga**

**gb:MG310229 BtCoV/20150819LFU_** **gctaatggcaacaaaattgttga**

**gb:MG817484 BtCoV/20141103SRP** **gctaatggcaacaaaattgttga**

**gb:MG252869 BtCoV/20160303FEK_** **gctaatggcaacaagattgttga**

**gb:MG252872 BtCoV/20160304FEK** **gctaatggcaacaagattgttga**

**gb:MG252873 BtCoV/20160304FEK_** **gctaatggcaacaagattgttga**

**gb:MG310226 BtCoV/20150105CGR_** **gctaatggcaacaagatcgttga**

**gb:MG817485 BtCoV/20150105CGR_** **gctaatggcaacaagatcgttga**

**gb:MG817486 BtCoV/20150105CGR_** **gctaatggcaacaagatcgttga**

**gb:MG205591 BtCoV/20150106CDK_** **gctaatggcaacaagattgttga**

**gb:MG252862 BtCoV/20150919CDK_** **gctaatggcaacaagattgttga**

**gb:MG817496 BtCoV/20150919CDK2** **gctaatggcaacaagattgttga**

**gb:MG252859 BtCoV/20150920CGC_** **gctaatggcaacaagattgttga**

**gb:MG817488 BtCoV/20150107CGC_** **gctaatggcaacaagattgttga**

**gb:MG817494 BtCoV/20150920CGC_** **gctaatggcaacaagattgttga**

**gb:MG252864 BtCoV/20150108CCK_** **gctaatggcaacaagattgttga**

**KC776174.1 Human MERS/CoV 2c J** **gctaatggcaacaagattgttga**

**MG923474.1 MERS-CoV camel/Nige** **gctaacggcaacaagattgttga**

**gb:KF500943 BtCoV/Pipistrellus** **actaatggcaacaaaattgttga**

**gb:KF500944 BtCoV/Pipistrellus** **actaatggcaacaaaattgttga**

**gb:KF500942 BtCoV/Pipistrellus** **actaatggcaacaaaattgttga**

**gb:KF500946 BtCoV/Pipistrellus** **actaatggcaacaaaattgttga**

**gb:KF500947 BtCoV/Pipistrellus** **actaatggcaacaaaattgttga**

**gb:HQ184059 BatCoV/H.sav/J/Spa** **actaatggcaacaaaattgttga**

**gb:KT717386 BatCoV Eum.gla/242** **actaatggcaacaaaattgttga**

**gb:GQ259977 BtCoV P.pipi/VM314** **actaacggcaacaagattgtcga**

**gb:KX285197 BtCoV/CHN/HKU5/PRE** **gctaatggcaacactattgttga**

**gb:KX285199 BtCoV/CHN/HKU5/PRE** **gctaatggcaacactattgttga**

**gb:KX285200 BtCoV/CHN/HKU5/PRE** **gctaatggcaacactattgttga**

**gb:HQ184062 BtCoV/E.isa/M/Spai** **gctaatggcaacaagattgttga**

**gb:KC522038 BtCoV/HKN/Tylonyct** **gctaatggtaatactatcataga**

**gb:KC522039 BtCoV/HKN/Tylonyct** **gctaatggtaatactatcataga**

**gb:KC522047 BtCoV/HKN/Tylonyct** **gctaatggtaatactatcataga**

**gb:KC522042 BtCoV/HKN/Tylonyct** **gctaatggtaatactatcataga**

**gb:KC522043 BtCoV/HKN/Tylonyct** **gctaatggtaatactatcataga**

**gb:KC522036 BtCoV/HKN/Tylonyct** **gctaatggtaatactatcataga**

**gb:KC522037 BtCoV/HKN/Tylonyct** **gctaatggtaatactatcataga**

**gb:KX447563 BatSARS/CHN/HKU3/1** **actgatggtaataagattgctga**

**gb:KX447564 BatSARS/CHNHKU3/15** **actgatggtaataagattgctga**

**gb:MG772855 BtCoV/CHN/SARS-lik** **actgatggtaataagattgctga**

**gb:MG772880 BtCoV/CHN/SARS-lik** **actgatggtaataagattgctga**

**gb:MG772855 SARS-like bat-SL-C** **actgatggtaataagattgctga**

**gb:MG772883 BtCoV/CHN/SARS-lik** **actgatggtaataagattgctga**

**gb:MG772862 BtCoV/CHNSARS-like** **actgatggtaataagattgctga**

**gb:MG772879 SARS-like bat-SL-C** **actgatggtaataagattgctga**

**gb:MG772858 BtCoV/CHN/SARS-lik** **actgatggtaataagattgctga**

**gb:MG772859 SARS-like bat-SL-C** **actgatggtaataagattgctga**

**gb:MG772858 SARS-like bat-SL-C** **actgatggtaataagattgctga**

**gb:KX285125 BatSARS/CHN/HKU3/P** **actgatggtaataagattgctga**

**gb:KX285125 BtCoV/SARS/CHN/HKU** **actgatggtaataagattgctga**

**gb:MG772861 SARS-like bat-SL-C** **actgatggtaataagattgctga**

**gb:MG772852 BtCoV/CHN/SARS-lik** **actgatggtaataagattgctga**

**gb:MG772875 BtCoV/CHNSARS-like** **actgatggtaataagattgctga**

**gb:MG772874 BtCoV/CHN/SARS-lik** **actgatggtaataagattgctga**

**gb:MG772865 BtCoV/CHN/SARS-lik** **actgatggtaataagattgctga**

**gb:MG772863 BtCoV/CHN/SARS-lik** **actgatggtaataagattgctga**

**gb:MG772891 BtCoV/CHN/SARS-lik** **actgatggtaataagattgctga**

**gb:MG772891 SARS-like bat-SL-C** **actgatggtaataagattgctga**

**gb:MG772890 BtCoV/CHN/SARS-lik** **actgatggtaataagattgctga**

**gb:MG772890 Bat SARS-like bat-** **actgatggtaataagattgctga**

**gb:MG772854 BtCoV/CHN/SARS-lik** **actgatggtaataagattgctga**

**gb:MG772854 SARS-like bat-SL-C** **actgatggtaataagattgctga**

**gb:MG772849 SARS-like bat-SL-C** **actgatggtaataagattgctga**

**gb:MG772848 SARS-like bat-SL-C** **actgatggtaataagattgctga**

**gb:MG772884 BtCoV/CHN/SARS-lik** **actgatggtaataagattgctga**

**gb:KY423420 BtCoV/FRA_EPI1_387** **actgatggtaataaaattgctga**

**gb:KY423421 BtCoVFRA_EPI1_3871** **actgatggtaataaaattgctga**

**gb:KY423431 BtCoV/FRA_EPI1_387** **actgatggtaataaaattgctga**

**gb:KY423422 BtCoV/FRA_EPI1_387** **actgatggtaataaaattgctga**

**gb:KY423428 BtCoV/FRA_EPI1_388** **actgatggtaataaaattgctga**

**gb:KY423425 BtCoV/FRA_EPI1_388** **actgatggtaataaaattgctga**

**gb:KY423430 BtCoV/FRA_EPI1_389** **actgatggtaataaaattgctga**

**gb:KY423424 BtCoV/FRA_EPI1_389** **actgatggtaataaaattgctga**

**gb:KY423432 BtCoV/FRA_EPI1_390** **actgatggtaataaaattgctga**

**gb:KY423419 BtCoV/FRA_EPI1_394** **actgatggtaataaaattgctga**

**gb:KY423427 BtCoV/FRA_EPI1_400** **actgatggtaataaaattgctga**

**gb:KY423417 BtCoV/FRA_EPI1_401** **actgatggtaataaaattgctga**

**gb:KY423397 FRA_EPI1_3875_3E_P** **actgatggtaataaaattgctga**

**gb:KY423398 BtCoV/FRA_EPI1_388** **actgatggtaataaaattgctga**

**gb:KY423399 BtCoV/FRA_EPI1_393** **actgatggtaataaaattgctga**

**gb:KY423411 BtCoV/FRA_EPI1_394** **actgatggtaataaaattgctga**

**gb:KY423400 BtCoV/FRA_EPI1_396** **actgatggtaataaaattgctga**

**gb:KY423396 BtCoV/FRA_EPI1_400** **actgatggtaataaaattgctga**

**gb:KY423395 BtCoV/FRA_EPI1_401** **actgatggtaataaaattgctga**

**gb:KY502395 SARS-rel/BtCoV/Rhi** **actgatggtaataaaattgctga**

**gb:KY423402 BtCoV/FRA_EPI1_387** **actgatggtaataaaattgctga**

**gb:KY423403 BtCoV/FRA_EPI1_Rhf** **actgatggtaataaaattgctga**

**gb:KY423388 BtCoV/FRA_EPI1_Rhf** **actgatggtaataaaattgctga**

**gb:KY423389 BtCoV/FRA_EPI1_Rhf** **actgatggtaataaaattgctga**

**gb:KY423390 BtCoV/FRA_EPI1_Rhf** **actgatggtaataaaattgctga**

**gb:KY423394 BtCoV/FRA_EPI1_Rhf** **actgatggtaataaaattgctga**

**gb:KY423392 BtCoV/FRA_EPI1_Rhf** **actgatggtaataaaattgctga**

**gb:KY423391 BtCoV/FRA_EPI1_Rhf** **actgatggtaataaaattgctga**

**gb:KY423386 BtCoV/FRA_EPI1_Rhf** **actgatggtaataaaattgctga**

**gb:KY423412 BtCoV/SPA_EPI1_Rhf** **actgatggtaataaaattgctga**

**gb:KY423375 BtCoV/FRA_EPI1_Rhf** **actgatggtaataaaattgctga**

**gb:KY423413 BtCoV/SPA_EPI1_Rhf** **actgatggtaataaaattgctga**

**gb:KY423433 BtCoV/FRA_EPI1_396** **actgatggtaataaaattgctga**

**gb:KY423437 BtCoV/FRA_EPI1_397** **actgatggtaataaaattgctga**

**gb:KY423438 BtCoV/FRA_EPI1_396** **actgatggtaataaaattgctga**

**gb:KC633214 BtCoV/Rhi_hip/R13-** **actgatggtaataagattgcaga**

**gb:KC633213 BtCoV/Rhi_hip/R46-** **actgatggtaataagattgcaga**

**gb:KC633212 BtCoV/Rhi_hip/R7-0** **actgatggtaataagattgcaga**

**gb:KC633209 BtCoV/Rhi_hip/R8-0** **actgatggtaataagattgcaga**

**gb:KC633210 BtCoV/Rhi_hip/R77-** **actgatggtaataagattgcagg**

**FJ710047.1 BtCoV/Ghana Kwam/20** **actaatggaaacaagattgtaga**

**FJ710054.1 BtCoV Hipposideros/** **actaatggaaacaagattgtaga**

**gb:HQ166910 ZBCoV**  **acaaatggaaacaaaattgtaga**

**gb:EU834950 BtCoV R.aur/Austra** **gtaaatggcaacgctatttcaga**

**KP112152.1 HuCoV HKU1/HCOV/KEN** **tgtaatggccataagattgaaga**

**KP112168.1 HuCoV/OC43 HCOV/KEN** **tgcaatggcaataagattgaaga**

**KP112167.1 UNVERIFIED: HuCoV/O** **tgcaatggcaataagattgaaga**

**KP112165.1 HuCoV/OC43 HCOV/KEN** **tgcaatggcaataagattgaaga**

**KP112163.1 HuCoV OC43 HCOV/KEN** **tgcaatggcaataagattgaaga**

**KP112161.1 HuCoV OC43 HCOV/KEN** **tgcaacggcaacaaaattgaaga**

**KC886322.1 BatCoV P.davyi49/Me** **gcagatggcaataagatctctaa**

**MG310257.1 alpha BtCoV/2014011** **attgattctaatacgtgcaacaa**

**MG817498.1 alpha BtCoV/2015010** **attgattccaatacatgcaacaa**

**MG310246.1 alpha BtCoV/2015010** **attgattccaatacgtgcaacaa**

**MG844332.1 alpha BtCoV/2014092** **attgattccaatacatgcaacaa**

**gb:GQ259961 BtCoV N.noc/VM176/** **attgattctaatacctgtaacaa**

**gb:GQ259968 BtCoV M.das/VM105/** **gttgatagtaacacttgtaacaa**

**gb:GQ259969 BtCoV M.das/VM62/N** **gttgatagtaacacttgtaacaa**

**gb:GQ259970 BtCoV M.das/VM73/N** **gttgatagtaacacttgtaacaa**

**gb:GQ259965 BtCoV M.das/VM3/NL** **gttgatagtaacacttgtaacaa**

**gb:GQ259966 BtCoV M.das/VM34/N** **gttgatagtaacacttgtaacaa**

**gb:GQ259971 BtCoV M.dau/VM222/** **gtcgatagtaacacttgtaacaa**

**gb:GQ259973 BtCoV M.dau/VM361/** **gttgatagtaacacttgtaacaa**

**MK603153.1 alpha BtCoV RIBSP-K** **gtggacagtaataactgtaacaa**

**MK603159.1 alpha BtCoV RIBSP-K** **gtggacagtaataactgtaacaa**

**MK603152.1 alpha CoV RIBSP-KZ-** **gtggacagtaataactgtaacaa**

**MK603160.1 alpha RIBSP-KZ-BatC** **gtggacagtaataactgtaacaa**

**MK603156.1 BtCoV alpha RIBSP-K** **gtggacagtaataactgtaacaa**

**MK603157.1 BtCoV alpha RIBSP-K** **gtggacagtaataattgtaacaa**

**MK603150.1 BtCoV alpha RIBSP-K** **gtggacagtaataactgtaacaa**

**gb:GQ259976 BtCoV M.das/VM2/NL** **gtcgacagtaataattgtaacaa**

**MG310240.1 alpha BtCoV/2015092** **gttaatagtaacacttgcaacaa**

**MG310244.1 alpha BtCoV/2015081** **gtggacagtaatacatgcaataa**
