# Supplementary material for: Evolutionary dynamics and geographic dispersal of beta coronaviruses in African bats
Source: PeerJ. 2020 Nov 26;8:e10434. doi: 10.7717/peerj.10434 (PMC7700737; doi:10.7717/peerj.10434)
Supplement: Supplemental Information 3 [file peerj-08-10434-s003.docx]

SUPPLIMENTARY TABLE 1

| No | Isolate Name | Year | Host | Country | Accession No |
| --- | --- | --- | --- | --- | --- |
| 1 | BatCoV/NIG/2011/13RS362-1 | 2011 | Bat | Nigeria | KU131210 |
| 2 | BatCoV/NGR/2011/13RS362-32 | 2011 | Bat | Nigeria | KU131211 |
| 3 | BatCoV/NGR/2011/13RS362-53 | 2011 | Bat | Nigeria | KU131212 |
| 4 | BatCoV/NGR/E.heivum/13RS362-59 | 2011 | Bat | Nigeria | KU131213 |
| 5 | BatCoV/NGR/E.heivum/13RS362-62 | 2011 | Bat | Nigeria | KU131214 |
| 6 | BatCoV/NGR/E.heivum/13RS452-71 | 2011 | Bat | Nigeria | KU131215 |
| 7 | BtCoV/20161014_DC172/Neoromicia/RSA | 2016 |  | South Afr | MG193617 |
| 8 | BtCoV/20150106CDK_NC1/Neoromicia/RSA | 2016 | Bat | South Afr | MG205591 |
| 9 | BtCoV/20150720ABA_NC1/Neoromicia/RSA | 2015 |  | South Afr | MG205593 |
| 10 | BtCoV/20150816HFP_NC1/Neoromicia/RSA | 2015 |  | South Afr | MG205595 |
| 11 | BtCoV/20150816HFP_NC3/Neoromicia/RSA | 2015 |  | South Afr | MG205596 |
| 12 | BtCoV/20150816HFP_PH1nc/Neoromicia/RSA | 2015 |  | South Afr | MG205597 |
| 13 | BtCoV/20150920CGC_NC2/Neoromicia/RSA | 2015 |  | South Afr | MG252859 |
| 14 | BtCoV/20150919CDK_NC10/Neoromicia/RSA | 2015 |  | South Afr | MG252862 |
| 15 | BtCoV/20150108CCK_NC3/Neoromicia/RSA | 2015 |  | South Afr | MG252864 |
| 16 | BtCoV/20160303FEK_NC1/Neoromicia/RSA | 2016 |  | South Afr | MG252869 |
| 17 | BtCoV/20160304FEK_NC1/Neoromicia/RSA | 2016 |  | South Afr | MG252872 |
| 18 | BtCoV/20160304FEK_NC2/Neoromicia/RSA | 2016 |  | South Afr | MG252873 |
| 19 | BtCoV/20161014_DC166/Neoromicia/RSA | 2016 |  | South Afr | MG252876 |
| 20 | BtCoV/20161014_DC_168/RSA | 2016 |  | South Afr | MG310223 |
| 21 | BtCoV/20141022HBI_PH10nc/Neoromicia | 2014 |  | South Afr | MG310225 |
| 22 | BtCoV/20150105CGR_NC2/Neoromicia/RSA | 2014 |  | South Afr | MG310226 |
| 23 | BtCoV/20150819LFU_PH2nc/Neoromicia/RSA | 2015 |  | South Afr | MG310229 |
| 24 | BtCoV/20161011_DC170/Neoromicia | 2016 |  | South Afr | MG310233 |
| 25 | BtCoV/20150816HFP_NC2/Neoromicia/RSA | 2015 |  | South Afr | MG310243 |
| 26 | BtCoV/20150720ABA_PH1nc/Neoromicia | 2015 |  | South Afr | MG310245 |
| 27 | BtCoV/20140127ABA_PH8nc/Neoromicia | 2014 |  | South Afr | MG817483 |
| 28 | BtCoV/20141103SRP_NC3/Neoromicia | 2014 |  | South Afr | MG817484 |
| 29 | BtCoV/20150105CGR_NC1/Neoromicia/RSA | 2015 |  | South Afr | MG817485 |
| 30 | BtCoV/20150105CGR_NC3/Neoromicia/RSA | 2015 |  | South Afr | MG817486 |
| 31 | BtCoV/20150107CGC_NC3/Neoromicia/RSA | 2015 |  | South Afr | MG817488 |
| 32 | BtCoV/20150920CGC_NC9/Neoromicia/RSA | 2015 |  | South Afr | MG817494 |
| 33 | BtCoV/20150919CDK2_NC2/Neoromicia | 2015 |  | South Afr | MG817496 |
| 34 | PREDICT-GVF-CM-ECO70514/CMR/2013 | 2013 | Bat | Cameroun | KX284999 |
| 35 | PREDICT-GVF-CM-ECO70516/2013/CMR | 2013 |  | Cameroun | KX285001 |
| 36 | PREDICT-GVF-CM-ECO70521/2013 | 2013 |  | Cameroun | KX285006 |
| 37 | PREDICT-GVF-CM-ECO70521/2013 | 2013 |  | Cameroun | KX285007 |
| 38 | PREDICT-GVF-CM-ECO70527/2013/CMR | 2013 |  | Cameroun | KX285008 |
| 39 | PREDICT-GVF-CM-ECO70527/2013/CMR | 2013 |  | Cameroun | KX285009 |
| 40 | PREDICT-GVF-CM-ECO70536/2013 | 2013 |  | Cameroun | KX285012 |
| 41 | PREDICT-GVF-CM-ECO70591/2013/CMR | 2013 |  | Cameroun | KX285023 |
| 42 | PREDICT-GVF-CM-ECO70592/CMR/2013 | 2013 | Bat | Cameroun | KX285024 |
| 43 | PREDICT-GVF-CM-ECO70592 | 2013 |  | Cameroun | KX285025 |
| 44 | PREDICT-130518Bt34/RWD/2013 | 2013 |  | Rwanda | KX285427 |
| 45 | PREDICT-130518Bt3/RWD/2013 | 2013 |  | Rwanda | KX285428 |
| 46 | PREDICT-130518Bt35/RWD/2013 | 2013 |  | Rwanda | KX285429 |
| 47 | PREDICT-140403Bt16/RWD/2014 | 2014 |  | Rwanda | KX285431 |
| 48 | PREDICT-CD115124A/DRC/2012 | 2012 |  | DRC | KX285070 |
| 49 | PREDICT-CD115222/DRC/2012 | 2012 |  | DRC | KX285071 |
| 50 | PREDICT-CD115912/DRC/2014 | 2014 |  | DRC | KX285075 |
| 51 | PREDICT-CD115914/drc/2014 | 2014 |  | DRC | KX285076 |
| 52 | PREDICT-CD115937/DRC/2014 | 2014 |  | DRC | KX285077 |
| 53 | PREDICT-CD115938/drc/2014 | 2014 |  | DRC | KX285078 |
| 54 | PREDICT-CD115941/DRC/2014 | 2014 |  | DRC | KX285080 |
| 55 | PREDICT-CD115943/DRC/2014 | 2014 |  | DRC | KX285081 |
| 56 | PREDICT-CD115947/DRC/2014 | 2014 |  | DRC | KX285082 |
| 57 | PREDICT-CD115956/DRC/2014 | 2014 |  | DRC | KX285085 |
| 58 | PREDICT-CD115975/DRC/2014 | 2014 |  | DRC | KX285086 |
| 59 | PREDICT-CD116004/DRC/2014 | 2014 |  | DRC | KX285087 |
| 60 | PREDICT-CD116015/DRC/2014 | 2014 |  | DRC | KX285088 |
| 61 | PREDICT-CD116096/DRC/2014 | 2014 |  | DRC | KX285099 |
| 62 | PREDICT-CD116102/DRC/2014 | 2014 |  | DRC | KX285101 |
| 63 | PREDICT-GVF-CM-ECO70379/CMR/2013 | 2013 | Bat | Cameroun | KX284989 |
| 64 | PREDICT-GVF-CM-ECO70379/CMR | 2013 | Bat | Cameroun | KX284990 |
| 65 | PREDICT-GVF-CM-ECO70509/CMR/2013 | 2013 |  | Cameroun | KX284994 |
| 66 | PREDICT-GVF-CM-ECO70509/CMR/2013 | 2013 |  | Cameroun | KX284993 |
| 67 | PREDICT-CD116101/DRC/2014 | 2014 |  | DRC | KX285100 |
| 68 | PREDICT-GVF-CM-ECO70332/CMR | 2013 |  | Cameroun | KX284986 |
| 69 | PREDICT-GVF-CM-ECO70332/CMR/2013 | 2013 |  | DRC | KX284987 |
| 70 | PREDICT-CD116103/DRC/2014 | 2014 |  | DRC | KX285102 |
| 71 | PREDICT-CD116105/DRC/2014 | 2014 |  | DRC | KX285103 |
| 72 | PREDICT-CD116107/DRC/2014 | 2014 |  | DRC | KX285105 |
| 73 | PREDICT-GVF-CM-ECO05710/CMR | 2010 |  | Cameroun | KX284951 |
| 74 | PREDICT-GVF-CM-ECO06214/CMR | 2011 |  | Cameroun | KX284954 |
| 75 | PREDICT-GVF-CM-ECO06409/CMR | 2013 |  | Cameroun | KX284957 |
| 76 | PREDICT-GVF-CM-ECO06417/CMR | 2013 |  | Cameroun | KX284958 |
| 77 | PREDICT-GVF-CM-ECO70284/CMR | 2013 |  | Cameroun | KX284985 |
| 78 | BtCoV/MDG/ANK036F/2010 | 2010 |  | Madagascar | KP696742.1 |
| 79 | BtCoV/MDG/ BEM073F/2010 | 2010 |  | Madagascar | KP696744.1 |
| 80 | BtCoV/MDG/ANK081F/2010 | 2010 |  | Madagascar | KP696747.1 |
| 81 | BtCoV/MDG/BEM077F | 2010 |  | Madagascar | KP696746.1 |
| 82 | BtCoV/MDG/BEM074F/2010 |  |  | Madagascar | KP696745.1 |
| 83 | BtCoV/KEN/E. heivum/BtKY110 | 2007 |  | Kenya | GU065384.1 |
| 84 | BtCoV/KEN/E. heivum/BtKY98 | 2007 |  | Kenya | GU065442.1 |
| 85 | Bt CoV/KEN/E. heivum/BtKY51 | 2007 |  | Kenya | GU065395.1 |
| 86 | BtCoV/KEN/E.heivum/BtKY103 | 2007 |  | Kenya | GU065437.1 |
| 87 | BtCoV/KEN/H.heivum/BtKY93 | 2007 | Bat | Kenya | GU065437.1 |
| 88 | BtCoV/KEN/E.heivum/BtKY104 | 2007 | Bat | Kenya | GU065378.1 |
| 89 | BtCoV/KEN/E.heivum/BtKY88 | 2007 | Bat | Kenya | GU065432.1 |
| 90 | BtCoV/KEN/E.heivum/BtKY87 | 2007 | Bat | Kenya | GU065431.1 |
| 91 | BtCoV/HKU5/PREDICT.EHA156.12-NL13847 | 2013 | Bat | China | KX285197 |
| 92 | BtCoV/HKU5/PREDICT-EHA156.12.NL13849 | 2013 | Bat | China | KX285199 |
| 93 | BtCoV/HKU5/PREDICT.EHA156-12.NL13856 |  | Bat | China | KX285200 |
| 94 | BatSARS/CHN/HKU3/152075 | 2015 | Bat | China | KX447563 |
| 95 | BatSARS/CHNHKU3/152090 | 2015 | Bat | China | KX447564 |
| 96 | BatSARS/ HKU3/ -EHA-156-11-AH-SXD002 | 2012 | Bat | China | KX285125 |
| 97 | BtCoV/CHN/SARS-like bat-SL-CoVDXC33 | 2015 | Bat | China | MG772858 |
| 98 | BtCoV/CHN/SARS-like bat-SL-CoVDXC49 | 2015 | Bat | China | MG772855 |
| 99 | BtCoV/CHN/SARS-like bat-SL-CoVDXC53 | 2015 | Bat | China | MG772854 |
| 100 | BtCoV/CHN/SARS-like bat-SL-CoVDXC59 | 2015 | Bat | China | MG772852 |
| 101 | BtCoV/CHNSARS-like bat-SL-CoVZ2_65 | 2013 | Bat | China | MG772875 |
| 102 | BtCoV/CHN/SARS-like bat-SL-CoVZ2_67 | 2013 | Bat | China | MG772874 |
| 103 | BtCoV/CHN/SARS-like bat-SL-CoVZ2_1 | 2013 | Bat | China | MG772891 |
| 104 | BtCoV/CHN/SARS-like bat-SL-CoVZ2_106 | 2013 | Bat | China | MG772865 |
| 105 | BtCoV/CHN/SARS-like bat-SL-CoVZ2_11 | 2013 | Bat | China | MG772884 |
| 106 | BtCoV/CHN/SARS-like bat-SL-CoVZ2_110 | 2015 | Bat | China | MG772863 |
| 107 | BtCoV/CHNSARS-like bat-SL-CoVZ2_113 | 2015 | Bat | China | MG772862 |
| 108 | BtCoV/CHN/SARS-like bat-SL-CoVZ2_16 | 2015 | Bat | China | MG772883 |
| 109 | BtCoV/CHN/SARS-like bat-SL-CoVZ2_2 | 2015 | Bat | China | MG772890 |
| 110 | BtCoV/CHN/SARS-like bat-SL-CoVZ2_45 | 2015 | Bat | China | MG772880 |
| 111 | BtCoVSARS/ HKU3/ EHA156-11AH-SXD002 | 2013 | Bat | China | KX285125 |
| 112 | SARS-like bat-SL-CoVDXC2/2016 | 2016 | Bat | China | MG772861 |
| 113 | SARS-like bat-SL-CoVDXC29/2016 | 2016 | Bat | China | MG772859 |
| 114 | SARS-like bat-SL-CoVDXC49/2016 | 2016 | Bat | China | MG772855 |
| 115 | SARS-like bat-SL-CoVDXC53/2016 | 2016 | Bat | China | MG772854 |
| 116 | SARS-like bat-SL-CoVDXC66/2016 | 2016 | Bat | China | MG772849 |
| 117 | SARS-like bat-SL-CoVDXC73/2016/CHN | 2016 | Bat | China | MG772848 |
| 118 | SARS-like bat-SL-CoVZ2_1/2016/CHN | 2016 | Bat | China | MG772891 |
| 119 | Bat SARS-like bat-SL-CoVZ2_2/2016 | 2016 | Bat | China | MG772890 |
| 120 | SARS-like bat-SL-CoVZ2_46/2016/CHN | 2016 | Bat | China | MG772879 |
| 121 | BtCoV/HKN/HKU9/Rousettus leschenaulti | 2013 | Bat | China | MG762628 |
| 122 | BtCoV/HKN/HKU9/Rousettus leschenaulti | 2013 | Bat | China | MG762629 |
| 123 | BtCoV/HKN/HKU9/Rousettus leschenaulti | 2013 | Bat | China | MG762630 |
| 124 | BtCoV/HKN/HKU9/Rousettus leschenaulti | 2013 | Bat | China | MG762631 |
| 125 | BtCoV/HKN/HKU9/Rousettus leschenaulti | 2013 | Bat | China | MG762633 |
| 126 | BtCoV/HKN/HKU9/Rousettus leschenaulti | 2013 | Bat | China | MG762634 |
| 127 | BtCoV/HKN/HKU9/Rousettus leschenaulti | 2013 | Bat | China | MG762635 |
| 128 | BtCoV/HKN/HKU9/Rousettus leschenaulti | 2013 | Bat | China | MG762636 |
| 129 | BtCoV/HKN/HKU9/Rousettus leschenaulti | 2013 | Bat | China | MG762639 |
| 130 | BtCoV/HKN/HKU9/Rousettus leschenaulti | 2013 | Bat | China | MG762640 |
| 131 | BtCoV/HKN/HKU9/Rousettus leschenaulti | 2013 | Bat | China | MG762641 |
| 132 | BtCoV/HKN/HKU9/Rousettus leschenaulti | 2013 | Bat | Hong Kong | MG762643 |
| 133 | BtCoV/HKN/HKU9/Rousettus leschenaulti | 2013 | Bat | Hong Kong | MG762646 |
| 134 | BtCoV/HKN/HKU9/Rousettus leschenaulti | 2013 | Bat | Hong Kong | MG762650 |
| 135 | BtCoV/HKN/Tylonycteris/HKU4/4R |  | Bat | Hong Kong | KC522038 |
| 136 | BtCoV/HKN/Tylonycteris/HKU4/5R |  | Bat | Hong Kong | KC522039 |
| 140 | BtCoV/HKN/Tylonycteris/HKU4/14R |  | Bat | Hong Kong | KC522047 |
| 141 | BtCoV/HKN/Tylonycteris/HKU4/2R |  | Bat | Hong Kong | KC522036 |
| 142 | BtCoV/HKN/Tylonycteris/HKU4/3R |  | Bat | Hong Kong | KC522037 |
| 143 | BtCoV/HKN/Tylonycteris/HKU4/8R |  | Bat | Hong Kong | KC522042 |
| 144 | BtCoV/HKN/Tylonycteris/HKU4/9R |  | Bat | Hong Kong | KC522043 |
| 145 | BtCoV/MYS/PREDICT_CoV-24/PMW 00241 |  | Bat | Hong Kong | KX285051 |
| 146 | Bt/CoV/Philippines/Diliman1525G2 PSW00711 | 2008 | Bat | Philippines | KX285112 |
| 147 | BtCoV/Philippines/Diliman1525G2/PSW 00715 | 2008 | Bat | Philippines | KX285113 |
| 148 | BtCoV/Philippines/Diliman1525G2/PSW 00735 | 2008 | Bat | Philippines | KX285114 |
| 149 | BtCoV/FRA_EPI1_3870_2H_P30 |  | Bat | France | KY423420 |
| 150 | BtCoVFRA_EPI1_3871_3A_P30 |  | Bat | France | KY423421 |
| 151 | BtCoV/FRA_EPI1_3874_3D_P30 |  | Bat | France | KY423431 |
| 152 | FRA_EPI1_3875_3E_P30 |  | Bat | France | KY423397 |
| 153 | BtCoV/FRA_EPI1_3877_3G_P30 | 2014 | Bat | France | KY423402 |
| 154 | BtCoV/FRA_EPI1_3879_4A_P30 | 2014 | Bat | France | KY423422 |
| 155 | BtCoV/FRA_EPI1_3880_4B_P30 | 2014 | Bat | France | KY423398 |
| 156 | BtCoV/FRA_EPI1_3881_4C_P30 | 2013 | Bat | France | KY423428 |
| 157 | BtCoV/FRA_EPI1_3882_4D_P30 | 2014 | Bat | France | KY423425 |
| 158 | BtCoV/FRA_EPI1_3897_6C_P30 | 2014 | Bat | France | KY423430 |
| 159 | BtCoV/FRA_EPI1_3899_6E_P30 |  | Bat | France | KY423424 |
| 160 | BtCoV/FRA_EPI1_3900_6F_P30 |  | Bat | France | KY423432 |
| 161 | BtCoV/FRA_EPI1_3932_10F_P30 |  | Bat | France | KY423399 |
| 162 | BtCoV/FRA_EPI1_3940_11F_P30 |  | Bat | France | KY423419 |
| 163 | BtCoV/FRA_EPI1_3944_12B_P30 |  | Bat | France | KY423411 |
| 164 | BtCoV/FRA_EPI1_3965_2G_p31 |  | Bat | France | KY423400 |
| 165 | BtCoV/FRA_EPI1_3966_2H_p31 |  | Bat | France | KY423438 |
| 166 | BtCoV/FRA_EPI1_3968_3B_p31 |  | Bat | France | KY423433 |
| 167 | BtCoV/FRA_EPI1_3975_4A_p31 |  | Bat | France | KY423437 |
| 168 | BtCoV/FRA_EPI1_4000_7B_p31 |  | Bat | France | KY423396 |
| 169 | BtCoV/FRA_EPI1_4015_9A_p31 |  | Bat | France | KY423395 |
| 170 | BtCoV/FRA_EPI1_4005_7G_p31 |  | Bat | France | KY423427 |
| 171 | BtCoV/FRA_EPI1_4016_9B_p31 |  | Bat | France | KY423417 |
| 172 | BtCoV/FRA_EPI1_Rhfer.pool1_B11_P10 |  | Bat | France | KY423403 |
| 173 | BtCoV/FRA_EPI1_Rhfer17_A3_P10 |  | Bat | France | KY423388 |
| 174 | BtCoV/FRA_EPI1_Rhfer19_C3_P10 |  | Bat | France | KY423389 |
| 175 | BtCoV/FRA_EPI1_Rhfer1_A1_P10 |  | Bat | France | KY423390 |
| 176 | BtCoV/FRA_EPI1_Rhfer23_G3_P10 |  | Bat | France | KY423375 |
| 177 | BtCoV/FRA_EPI1_Rhfer39_G5_P10 |  | Bat | France | KY423394 |
| 178 | BtCoV/FRA_EPI1_Rhfer42_B6_P10 |  | Bat | France | KY423392 |
| 179 | BtCoV/FRA_EPI1_Rhfer57_A8_P10 |  | Bat | France | KY423391 |
| 180 | BtCoV/FRA_EPI1_Rhfer59_11B_P29 |  | Bat | France | KY423386 |
| 181 | BtCoV/Pipistrellus kuhlii/Italy/206645-27 | 2011 | Bat | Italy | KF500943 |
| 182 | BtCoV/Pipistrellus kuhlii/Italy/206645-29 |  | Bat | Italy | KF500944 |
| 183 | BtCoV/Pipistrellus kuhlii/Italy/206645-3 | 2011 | Bat | Italy | KF500942 |
| 184 | BtCoV/Pipistrellus kuhlii/Italy/206645-53 |  | Bat | Italy | KF500946 |
| 185 | BtCoV/Pipistrellus kuhlii/Italy/206645-54 |  | Bat | Italy | KF500947 |
| 186 | BtCoV/SPA_EPI1_Rhfer19_12B_P24 |  | Bat | Spain | KY423413 |
| 187 | BtCoV/SPA_EPI1_Rhfer25_6H_P24 |  | Bat | Spain | KY423412 |
| 188 | BtCoV/Rhi_hip/R13-08/SPA/2010 | 2010 | Bat | Spain | KC633214 |
| 189 | BtCoV/Rhi_hip/R46-03/SPA/2010 |  | Bat | Spain | KC633213 |
| 190 | BtCoV/Rhi_hip/R7-08/SPA/2010 |  | Bat | Spain | KC633212 |
| 191 | BtCoV/Rhi_hip/R77-02/SPA/2010 |  | Bat | Spain | KC633210 |
| 192 | BtCoV/Rhi_hip/R8-09/SPA/2010 |  | Bat | Spain | KC633209 |
| 193 | BtCoV/E.isa/M/Spain/2007 | 2007 | Bat | Spain | HQ184062 |
| 194 | BatCoV/H.sav/J/Spain/2007 | 2007 | Bat | Spain | HQ184059 |
| 195 | SARSr/BtCoV/Rhinolophus ferrumequinum/LUX | 2016 | Bat | Luxumburg | KY502395 |
| 196 | Human MERS/CoV 2c Jordan-N3/2012 | 2012 | Human | Jordan | KC776174.1 |
| 194 | MERS-CoV camel/Nigeria/NV1405 | 2016 | Camel | Nigeria | MG923474.1 |
| 195 | HuCoV HKU1/HCOV/KENYA | 2009 | Human | Kenya | KP112152.1 |
| 196 | HuCoV/OC43 HCOV/KENYA/022 |  | Human | Kenya | KP112168.1 |
| 197 | HuCoV/OC43 HCOV/KENYA/021 |  | Human | Kenya | KP112167.1 |
| 198 | HuCoV/OC43 HCOV/KENYA/019 |  | Bat | Kenya | KP112165.1 |
| 199 | HuCoV OC43 HCOV/KENYA/007/2010 | 2010 | Bat | Kenya | KP112161.1 |
| 200 | HuCoV OC43 HCOV/KENYA/017 | 2010 | Bat | Kenya | KP112163.1 |
| 201 | BtCoV/20140115AHHB_PSP1ncNeoromicia |  | Bat | South Afr | MG310257.1 |
| 202 | BtCoV/20140923LNR_NC7/12/Neoromicia |  | Bat | South Afr | MG844332.1 |
| 203 | BtCoV/20150106CDK_NC9/Neoromicia |  | Bat | South Afr | MG817498.1 |
| 204 | BtCoV/20150108CCK_NC7/Neoromicia |  | Bat | South Afr | MG310246.1 |
| 205 | BtCoV/20150815HFP_PH5nc/Neoromicia |  | Bat | South Afr | MG310244.1 |
| 206 | BtCoV RIBSP-KZ-BatCoV-11 |  | Bat | South Afr | MK603153.1 |
| 207 | alpha BtCoV RIBSP-KZ-BatCoV-62 |  | Bat | South Afr | MK603159.1 |
| 208 | CoV RIBSP-KZ-BatCoV-7 |  | Bat | South Afr | MK603152.1 |
| 209 | CoV RIBSP-KZ-BatCoV-7 |  | Bat | South Afr | MK603152.1 |
| 210 | alpha RIBSP-KZ-BatCoV-65 |  | Bat | South Afr | MK603160.1 |
| 211 | BtCoV alpha RIBSP-KZ-BatCoV-20 |  | Bat | South Afr | MK603157.1 |
| 212 | BtCoV alpha RIBSP-KZ-BatCoV-3 |  | Bat | South Afr | MK603150.1 |
| 213 | BtCoV alpha RIBSP-KZ-BatCoV-18 |  | Bat | South Afr | MK603156.1 |
| 214 | BtCoV/20150922CDK1_MN1/Miniopteru |  | Bat | South Afr | MG310240.1 |
| 215 | BtCoV N.noc/VM176/NLD/2007 | 2007 | Bat | Netherland | GQ259961 |
| 216 | BtCoV M.das/VM105/2006 | 2006 | Bat | Netherland | GQ259968 |
| 217 | BtCoV M.das/VM62/NLD | 2007 | Bat | Netherland | GQ259969 |
| 218 | BtCoV M.das/VM73/NLD | 2007 | Bat | Netherland | GQ259970 |
| 218 | BtCoV M.das/VM3/NLD | 2007 | Bat | Netherland | GQ259965 |
| 219 | BtCoV M.das/VM34/NLD |  | Bat | Netherland | GQ259964 |
| 220 | BtCoV M.das/VM222/NLD | 2007 | Bat | Netherland | GQ259971 |
| 221 | BtCoV M.das/VM361/NLD |  | Bat | Netherland | GQ259973 |
| 222 | BatCoV P.davyi49/Mexico/2012 | 2012 | Bat | Mexico | KC886322 |
| 223 | BtCoV R.aur/Australia/CoV000/2006 | 2006 | Bat | Australia | EU834950 |
| 224 | ZBtCoV |  | Bat | Nigeria | HQ166910 |
| 225 | BtCoV Hipposideros/GhanaKwam/22/2008 | 2008 | Bat | Ghana | FG710054.1 |
| 226 | BtCoV/Ghana Kwam/20/Hipposiderus/2008 | 2008 | Bat | Ghana | FG710047 |
| 227 | BatCoV Eum.gla/242/BRA/2013 | 2013 | Bat | Brazil | KT717386 |
| 228 | BtCoV P.pipi/VM314/NLD/2008 | 2008 | Bat | Netherland | GQ259977 |
|  |  |  |  |  |  |
